# Tracking motion kinematics and tremor with intrinsic oscillatory property of instrumental mechanics

**DOI:** 10.1002/btm2.10432

**Published:** 2022-10-22

**Authors:** Chun‐Lun Ni, Yi‐Ting Lin, Liang‐Yin Lu, Jia‐Huei Wang, Wen‐Chuan Liu, Sheng‐Han Kuo, Ming‐Kai Pan

**Affiliations:** ^1^ Department of Neurology Columbia University New York New York USA; ^2^ The Initiative for Columbia Ataxia and Tremor New York New York USA; ^3^ Department of Biochemistry and Molecular Biology Indiana University School of Medicine Indianapolis Indiana USA; ^4^ Molecular Imaging Center, National Taiwan University Taipei City Taiwan; ^5^ Department of Psychology National Taiwan University Taipei City Taiwan; ^6^ Institute of Biomedical Sciences, Academia Sinica Taipei City Taiwan; ^7^ Department and Graduate Institute of Pharmacology National Taiwan University College of Medicine Taipei City Taiwan; ^8^ Department of Medical Research National Taiwan University Hospital Taipei City Taiwan; ^9^ Cerebellar Research Center National Taiwan University Hospital, Yun‐Lin Branch Yun‐Lin Taiwan

**Keywords:** essential tremor, force plate, motion tracking, movement disorder

## Abstract

Tracking kinematic details of motor behaviors is a foundation to study the neuronal mechanism and biology of motor control. However, most of the physiological motor behaviors and movement disorders, such as gait, balance, tremor, dystonia, and myoclonus, are highly dependent on the overall momentum of the whole‐body movements. Therefore, tracking the targeted movement and overall momentum simultaneously is critical for motor control research, but it remains an unmet need. Here, we introduce the intrinsic oscillatory property (IOP), a fundamental mechanical principle of physics, as a method for motion tracking in a force plate. The overall kinetic energy of animal motions can be transformed into the oscillatory amplitudes at the designed IOP frequency of the force plate, while the target movement has its own frequency features and can be tracked simultaneously. Using action tremor as an example, we reported that force plate‐based IOP approach has superior performance and reliability in detecting both tremor severity and tremor frequency, showing a lower level of coefficient of variation (CV) compared with video‐ and accelerometer‐based motion tracking methods and their combination. Under the locomotor suppression effect of medications, therapeutic effects on tremor severity can still be quantified by dynamically adjusting the overall locomotor activity detected by IOP. We further validated IOP method in optogenetic‐induced movements and natural movements, confirming that IOP can represent the intensity of general rhythmic and nonrhythmic movements, thus it can be generalized as a common approach to study kinematics.

## 1 INTRODUCTION

Quantifying the kinematic details of motor behaviors is a foundation to study the biology of motor control and movement disorders. The kinematic profiles, such as position, velocity, or acceleration of various body parts, are highly dynamic in the timescale of millisecond. Therefore, current technology of motion tracking focuses on the high‐definition tracking of one limb or one targeted motion. However, many motor phenomena, such as reaching movement, walking, or balancing, involved complex coordinated movement beyond the limb being studied. For example, the cerebellum engages different functions for walking when two legs in human (or two sides of limbs in mice) move in the same versus different cycling speed.^1^, ^2^, ^3^, ^4^ This has been the foundation of cerebellar‐based motor learning and rehabilitation after cerebral insults.^3^, ^4^ Adding to the complexity, most of the movement disorders behave very differently when volitional movement is involved. The dystonia severity is exacerbated, known as “overflow phenomenon,” when the supported arm or trunk are also moving.^5^, ^6^ The intensity of tremor, which is an involuntary rhythmic movement, increases with bigger volitional movement.^7^, ^8^, ^9^, ^10^, ^11^, ^12^ There is a clear need for a technology to simultaneously track the motor kinematics of both targeted limb movement and overall motion.

To date, motion tracking depends on the camera for superior information of limb/body position, but it is limited by the data rate and restricted scenarios suitable for video capturing.^13^, ^14^ High‐speed camera array provides better temporal resolution, but the more stringent illumination and imaging depth limit their applications to the scenario for freely volitional movement, which is critical to the manifestation of movement disorders such as tremor, dystonia, and myoclonus, and complex natural behaviors such as balancing and turning. Novel tracking techniques, like fluoroscope and skin‐marker (optical marker) tracking system, provide further kinematics details to track specific motion but also face the limitations that traditional video methods suffer from.^15^, ^16^, ^17^, ^18^, ^19^ In high‐resolution scenarios, they are more sensitive to environmental settings and global motions, therefore limiting their application to head‐fixed or restrained settings. Another option is accelerometer‐based detection. Accelerometer has superior temporal resolution but is less capable of differentiating targeted movement from overall locomotor activity contributed by other body parts.^20^, ^21^, ^22^ On the other hand, ultrasound‐based motion tracking provides precise kinematics information for fine movements, but it is not designed to track free‐moving global movements.^23^ Besides, among the above‐mentioned tracking techniques, only the traditional video methods and the accelerometer method are well established and affordable at the same time. Collectively, a high‐precision, easy to use motion tracking system for movement disorders is still an unmet need.

Here, we introduce another method, intrinsic oscillatory property (IOP) of a harmonic oscillator, to measure overall momentum in a freely moving animal. Based on the basic mechanical principle of physics, intrinsic oscillation is a fundamental phenomenon existing in every rigid body attached to a spring. Any movement applied to the system will trigger the intrinsic oscillation, and the amplitude of this frequency‐specific vibration is proportional to the given force. Such system generates natural oscillatory frequency (*f*) shown in equation below, where *k* and *m* here represent spring constant and mass, respectively:

$$f=\frac{1}{2\pi }\sqrt{\frac{k}{m}}$$

**FIGURE 1:**
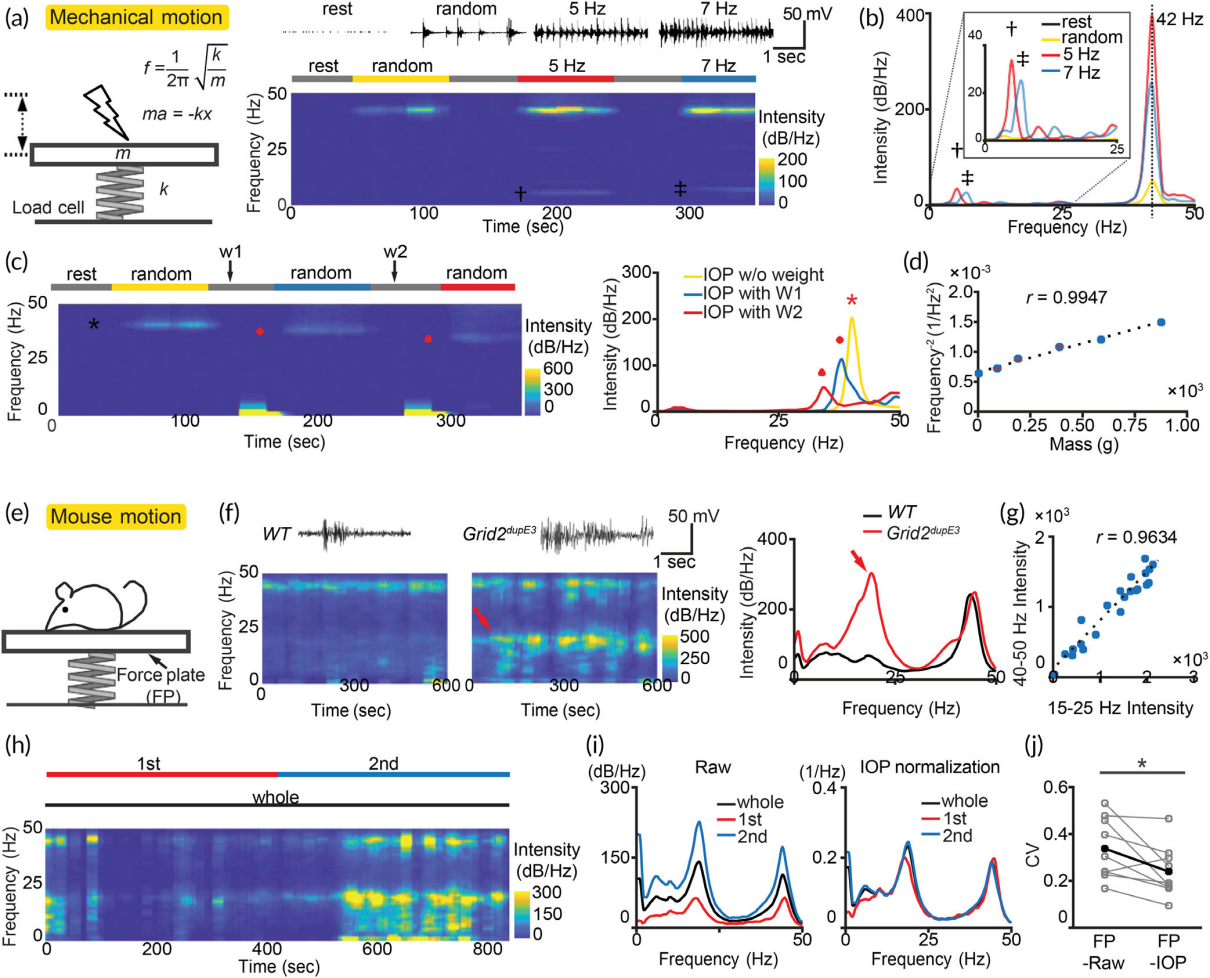
Distinguishable frequencies between rhythmic activity and intrinsic oscillations that reflect overall motion. (a) Scheme of force plate‐based motion detection with intrinsic oscillatory properties. The oscillatory frequency (*f*) of a force plate depends on system stiffness (*k*) and mass (*m*), but not displacement (*x*) or acceleration (*a*). (b) Mechanical tapping‐induced IOP (~42 Hz), dissociable from rhythmic tapping frequency (5 or 7 Hz). Bars above heatmap: analyzed periods. Dagger (**†**): 5 Hz. Double dagger (**††)**: 7 Hz. (c) IOP frequency tuning with different weights (W1 and W2). Asterisk (*), diamond, and triangle indicate frequencies with different masses. (d) Correlation of mass and the corresponding IOP frequency, consistent with the formula in Figure 1a. Error bar (SEM) is invisible if smaller than dot. (*n* = 8 per dot) 95% confidence interval: [0.9502, 0.9994]; *t*_4_ = 19.3484, *p* < 0.0001, *t*‐test. (e) Scheme of mouse motion detection. (f) Detection of *Grid2* ^*dupE3*^ mouse tremor (red arrow) with locomotion detected by IOP. (g) Correlation of tremor intensity and IOP. (*n* = 22 from a mouse) 95% confidence interval: [0.9124, 0.9849]; *t*_20_ = 16.0723, *p* < 0.0001, *t*‐test. (h, i) Reduction of *Grid2* ^*dupE3*^ tremor variation after normalization to IOP. Bars above heatmap: analyzed periods. (j) Reduction of coefficient of variation (CV) after IOP normalization. (*n* = 9 mice) *p < 0.05, Wilcoxon signed‐rank test

Thus, IOP could express in a force plate equipped with a single force transducer as the force reporter, pedestal and elastic component to generate intrinsic oscillations (Figure 1a). The kinetic energy of the animal can be transformed to the amplitudes at the intrinsic oscillatory frequency. Additionally, with proper tuning, a force plate can have a designed intrinsic oscillatory frequency that is separated from the kinematic frequency of the specific movement being studied. On this basis, a transducer can output both specific kinematics and overall locomotion in distinct frequency ranges. With the aid of this technique, it is possible to track the overall locomotion besides the targeted motor phenomenon. When these two kinds of activity are tracked properly, it is feasible to calibrate the measured intensity of targeted motion from the level of overall locomotion.

In this study, we first the IOP method with existing methods in a mouse model of essential tremor exhibiting pure action tremor.^24^ Action tremor is characterized by frequency‐specific movements during action,^7^, ^8^, ^25^ which is a biological feature independent of measuring methods. Hence, essential tremor can serve as an adequate model to compare the validity and reliability of motion‐tracking tools. Moreover, action tremor is the core symptom of essential tremor, the most common movement disorder that affects up to 20% of elderly population.^26^, ^27^ However, there is a clear unmet medical need for the technology of tremor measurement in animal trials. The action‐dependent feature of essential tremor impedes comparison between individuals or between tests within the same animal. The intensities of action tremor are highly modulated by the speed or overall momentum of volitional movement.^7^, ^8^, ^9^, ^10^, ^11^, ^12^ Specifically, less tremor is present under lower locomotor activity, making therapeutic tremor‐suppression indistinguishable from sedative effect. We thus performed head‐to‐head comparison of IOP with video‐ and accelerometer‐based methods^24^, ^28^, ^29^, ^30^ for tremor measurement in a mouse model.^24^

We also validated the IOP method for tracking motion kinematics beyond tremor, including optogenetic‐induced movement and nonrhythmic natural movement. IOP method is shown to be capable of capturing overall kinematics of an animal under artificially induced motion. It is also shown to represent the global natural movement. Collectively, the IOP method can be a useful tool for studying various motor phenomena. Adding to its superiority in tracking motion kinematics, its low‐cost and easy‐to‐use characteristics make it a potential novel approach to advance our understanding of motion phenomena and motion disorders.

## 2 RESULTS

### 2.1 Intrinsic oscillations of force plate reflecting both periodic and nonperiodic forces

We built a force plate with IOP (Figure 1a, formula). It is assembled by a basal plate, a loading cell, and a preamplifier (see Section 5 and Figure S1). To validate whether abovementioned physical property can be faithfully presented in the force plate and can be used in tremor recording, we first applied forces by mechanical tapping at 5 Hz, 7 Hz or nonperiodic random tapping (Figure 1a). During tapping, both periodic and nonperiodic forces generated intrinsic oscillations at ~42 Hz, reflecting the natural frequency in our system (Figure 1b). The oscillations went through fast attenuation and were not detectable during nontapping period. During periodic tapping, the force plate reliably detected the rhythmic motion at 5 Hz or 7 Hz (Figure 1b, magnified). The results suggest that IOP can be a reliable method for motion detection without compromising the ability to record the force rhythm. We also examined IOP with different masses on the force plate. The observed frequency was reduced when additional mass was applied on the plate (Figure 1c). Curve fitting on the frequency‐mass dataset (Figure 1d) was highly consistent with the mechanical principle of physics (Figure 1a, formula). Taken together, IOP in a force plate can reliably capture both rhythmic and nonrhythmic force, and the oscillatory frequency can be tuned by adjusting the mass of the system.

### 2.2 IOP‐based motion detection for tremor measurement and normalization

To examine whether IOP can be used for quantitative motion detection with high temporal resolution and accuracy, we next applied action tremor as the model for kinematic measurement (Figure 1e). We measured rhythmic activity in either wild‐type mice or homozygous *Grid2* ^*dupE3*^ mice, which had action tremor at 20 Hz.^24^ Intrinsic oscillations at 42 Hz were found in both wild‐type and *Grid2* ^*dupE3*^ mice, while the tremor spectrum with the evidenced peak at 20 Hz was only detected in *Grid2* ^*dupE3*^ mice (Figure 1f, red arrow). We next examined the correlation of intensity between intrinsic oscillations and tremor in *Grid2* ^*dupE3*^ mice (Figure 1g). There was a strong linear correlation between tremor intensity and intrinsic oscillatory intensity. As IOP reflects overall kinematic activity, this strong correlation is compatible with previous studies that tremor intensity is affected by overall locomotor activity.^7^, ^8^, ^9^, ^10^, ^11^, ^12^

Since *tremor severity* of a given animal is a state of the disease, it should be the same across trials and independent of the animal's locomotor activity, which modulates the *raw intensity* of action tremor (Figure 1g,h). In addition, a stable measurement for the state of tremor (tremor severity) is crucial to evaluate pharmacological responses of tremor. We then examined whether the tremor intensity normalized by IOP could be used as a more stable index for the disease state. Our results showed that *normalized tremor intensity* was more stable across the recording, providing a stable assessment of tremor (Figure 1i). To quantify the variation of raw tremor intensity versus normalized tremor intensity across time, we calculated the coefficient of variation (CV) of the tremor intensity during the first and second half periods from a given recording. Comparing CVs with or without normalization, IOP‐normalization significantly reduced the variation of tremor intensity related to locomotor activity (mean(SEM): FP‐Raw 0.34 (0.04), FP‐IOP 0.24 (0.04); Figure 1j).

Taken together, the IOP method can be a reliable method to measure overall momentum. Therefore, IOP can be used to normalize the action‐dependent variability of tremor and generates a more stable readout indicating activity‐adjusted *tremor severity*, which reflects a reliable proportion of tremor (tremor intensity) among overall motion (intrinsic oscillatory intensity).

### 2.3 Comparison of IOP with video‐based motion detection for tremor normalization

**FIGURE 2:**
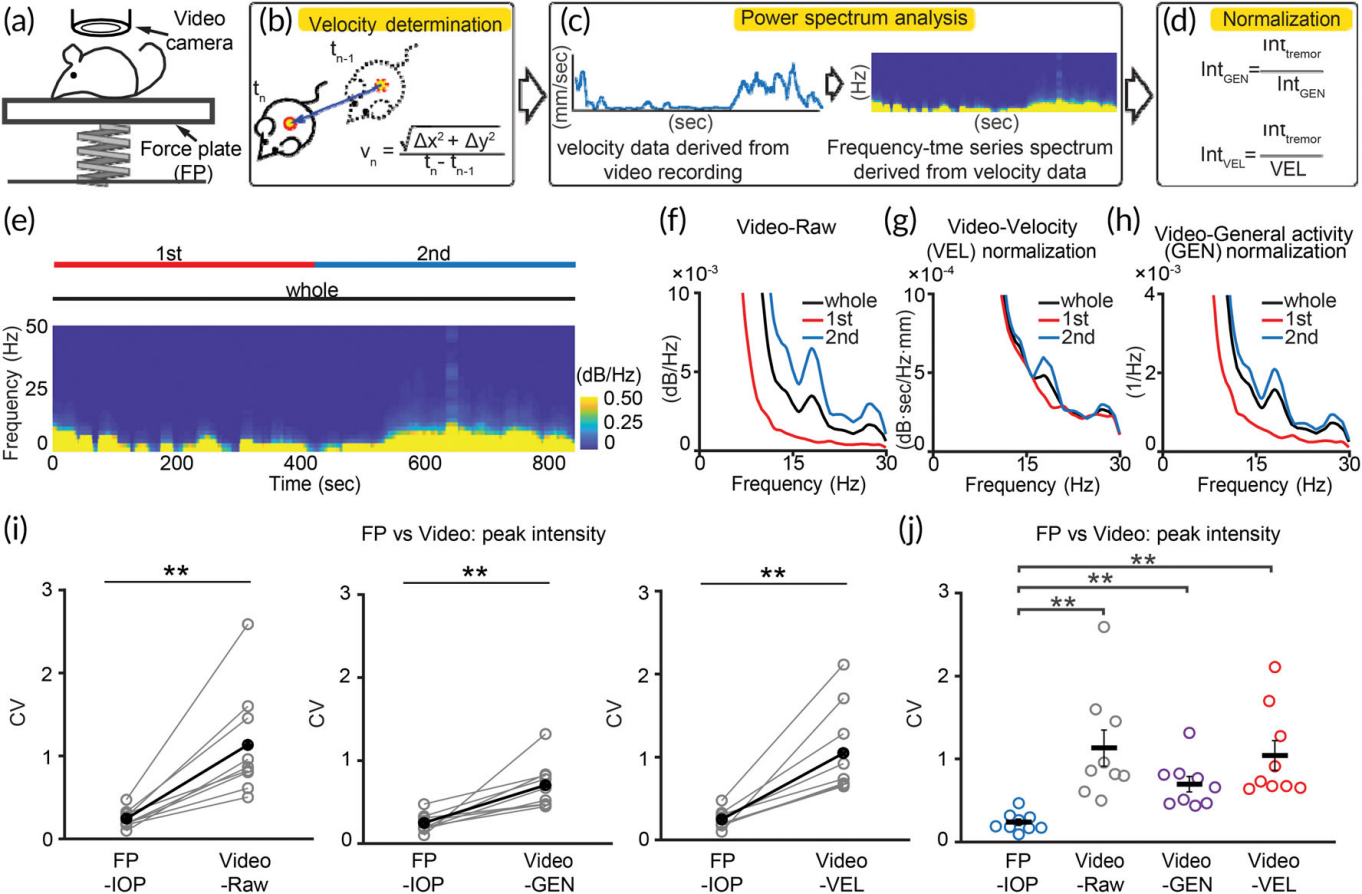
Comparison of video‐ and IOP‐based tremor measurements. (a) Scheme of *Grid2* ^*dupE3*^ motion detection by simultaneous video‐based and force plate (FP)‐IOP‐based recordings for head‐to‐head comparison. (b–d) Flowchart of data analysis. Velocity data were derived from video recording (panel b), followed by power spectrum analysis (panel c), and normalization (panel d) to general activity (GEN, overall intensity from 2 to 30 Hz) or moving velocity (VEL). (e–h) Representative power spectrum analysis and normalization of video‐based data. Bars above heatmap: analyzed periods. (i, j) Comparison of IOP‐ and video‐based methods. (*n* = 9 mice) **p < 0.01, Wilcoxon signed‐rank test. Black dots and horizontal black bars: mean. Black error bars: SEM

To evaluate the efficacy of IOP‐based normalization for tremor, we first compared it with video‐based tremor detection and normalization (Figure 2a–d). Frame‐by‐frame detection of *Grid2* ^*dupE3*^ mouse position in a 60 frame‐per‐second video allowed further analysis of mouse moving velocity (Movie S2), whereas spectral analysis of the moving velocity provided frequency‐specific information reflecting tremor intensity (Figure 2e,f). We normalized video‐based tremor profiles by either moving velocity (Figure 2g) or the integrated power between 2 and 30 Hz, reflecting the overall mouse motion (general activity, Figure 2h). By comparing the consistency of tremor frequency or normalized tremor severity, IOP‐based approach is significantly better than video‐based methods (mean(SEM): FP‐IOP 0.24 (0.04), Video‐Raw 1.13 (0.22), Video‐GEN 0.69 (0.09), Video‐VEL 1.04 (0.18); Figure 2i,j).

### 2.4 Comparison of IOP method with video‐assisted, accelerometer‐based tremor measurement

**FIGURE 3:**
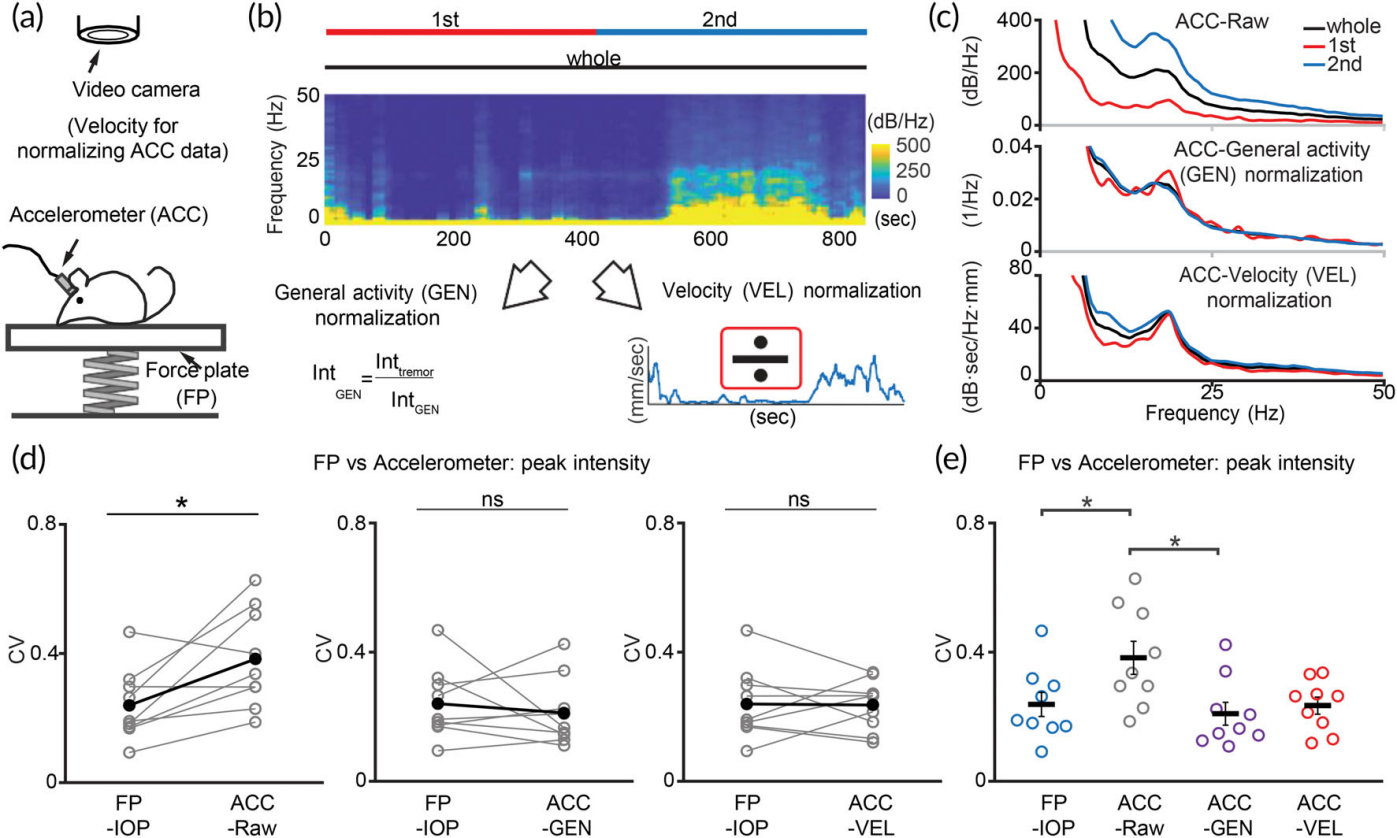
Comparison of accelerometer‐ and IOP‐based tremor measurements. (a) Scheme of *Grid2* ^*dupE3*^ mouse motion detection by simultaneous accelerometer (ACC)‐based, video‐based, and force plate (FP)‐IOP‐based recordings for head‐to‐head comparison. (b) Normalization of ACC‐based data. After power spectrum analysis, data were normalized to general activity (GEN, overall 2–50 Hz intensity) or moving velocity (VEL, derived from video data). (c) Representative frequency distributions of different time periods, before and after normalization. (d, e) Comparison of IOP‐ and ACC‐based methods. (*n* = 9 mice) *p < 0.05, Wilcoxon signed‐rank test. Black dots and horizontal black bars: mean. Black error bars: SEM

Similar to the force plate, accelerometer allows motion recording with high temporal precision (1 kHz in our settings), therefore provides better frequency resolution in terms of tremor measurement by frequency spectrum analysis. To compare IOP‐based tremor normalization with accelerometer‐based measurement, here we also applied two methods for normalization of accelerometer detection (Figure 3a,b). Accelerometer‐based general activity, similar to the video methods, was derived from the summation of spectral power across motion‐related frequencies (Figure 3c, ACC‐Raw, 2–50 Hz) of accelerometer‐detected frequency spectrum for normalization (Figure 3b,c, ACC‐General activity normalization). We also combined the video and accelerometer data by using video‐based moving velocity to obtain normalized accelerometer‐based spectral data (Figure 3b,c, ACC‐velocity activity normalization). When compared with pure video‐based methods, the consistency of accelerometer‐based measurement was significantly improved, either by normalization with accelerometer‐based general activity or by video‐based velocity measurement. (mean(SEM): ACC‐Raw 0.38 (0.05), ACC‐GEN 0.21 (0.04), ACC‐VEL 0.24 (0.03), Video‐Raw 1.13 (0.22), Video‐GEN 0.69 (0.09), Video‐VEL 1.04 (0.18); Figure S2). After normalization to either measurement, the consistency of tremor severity from accelerometer data seemed to reach the level of IOP‐based method (mean(SEM): FP‐IOP 0.24 (0.04), ACC‐Raw 0.38 (0.05), ACC‐GEN 0.21 (0.04), ACC‐VEL 0.24 (0.03); Figure 3d,e).

Although tremor intensity determined by accelerometer apparently exhibited consistency (Figure 3d,e), we noticed that there was a considerable background component in the frequency domain with exponential decay (Figure 3c). We therefore asked whether such background components affect the consistency calculation in the tremor severity derived from accelerometer‐based methods. Since CV is the ratio of the standard deviation to the mean, the existence of this background component will reduce the CV by increasing the mean and thus will cause the illusion of improved data consistency (Figure S3). Moreover, tremor peak frequencies determined by accelerometer‐based methods were more variable than the IOP method (mean(SEM): FP‐IOP 0.03 (0.002), ACC‐Raw 0.07 (0.01), ACC‐GEN 0.07 (0.01), ACC‐VEL 0.06 (0.01); Figure S4), suggesting the existence of considerable background may mislead the detection of tremor peak frequency, which is assigned to the local maximum of the spectrum (Figure S5). Therefore, subtraction of the background component should be performed to reveal the true variability of tremor intensity and frequency.

**FIGURE 4:**
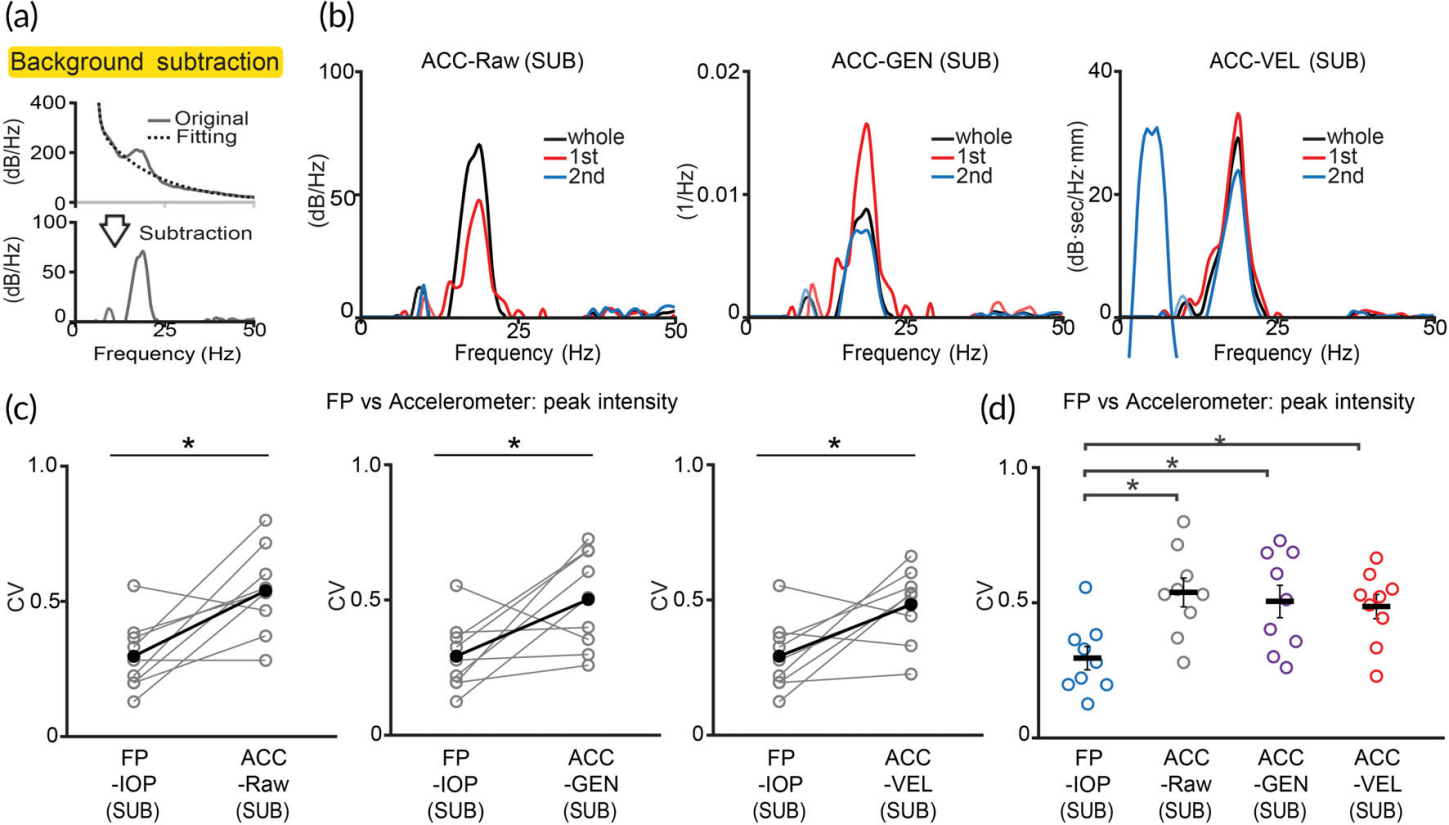
Comparison of accelerometer‐ and IOP‐based tremor measurements after background subtraction. (a) Subtraction of nontremor component. Background component computed by fitting nontremor portion of the spectrum was subtracted from the original spectrum to calibrate tremor signal (SUB). (b) Representative frequency distributions after background subtraction. Data were derived from Figure 3c. (c, d) Comparison of IOP‐ and ACC‐based methods after background subtraction. (*n* = 9 mice) *p < 0.05, Wilcoxon signed‐rank test. Black dots and horizontal black bars: mean. Black error bars: SEM

We therefore performed curve fitting to the nontremor portion of the spectral data (Figure 4a, original) and subtract it from the spectrum (Figure 4a, subtraction) in both accelerometer‐based data and IOP‐based data. After background subtraction, variability of detected tremor severity by all accelerometer‐based methods were increased (mean(SEM) with background, mean(SEM) without background: ACC‐Raw 0.38 (0.05), 0.54 (0.05); ACC‐GEN 0.21 (0.04), 0.51 (0.06); ACC‐VEL 0.24 (0.03), 0.49 (0.04); Figure 4b and Figure S6), reflecting the true variations of tremor severity. In this context, IOP method showed significant better consistency than accelerometer‐based methods. (mean(SEM): FP‐IOP(SUB) 0.30 (0.04), ACC‐Raw(SUB) 0.54 (0.05), ACC‐GEN(SUB) 0.51 (0.06), ACC‐VEL(SUB) 0.49 (0.04); Figure 4c,d).

Furthermore, background subtraction significantly improved the consistency of detected tremor frequency in accelerometer‐ and video‐based methods (mean(SEM) with background, mean(SEM) without background: ACC‐Raw 0.07 (0.01), 0.05 (0.005); ACC‐GEN 0.07 (0.01), 0.05 (0.005); ACC‐VEL 0.06 (0.01), 0.04 (0.004); Video‐Raw 0.14 (0.01), 0.10 (0.01); Video‐GEN 0.15 (0.01), 0.09 (0.01); Video‐VEL 0.15 (0.01), 0.09 (0.01); Figure S7), suggesting the background component has a negative effect on tremor frequency determination in these methods. Consistently, after subtracting the background component unrelated to tremor variability, the likelihood to identify local spectral maximum within the 15–25 Hz window (true tremor peak) was significantly increased (likelihood with background, likelihood without background: ACC‐Raw 0.95, 1; ACC‐GEN 0.95, 1; ACC‐VEL 0.95, 1; Video‐Raw 0.76, 0.94; Video‐GEN 0.76, 0.93; Video‐VEL 0.76, 0.94; Figure S8).

### 2.5 Validation of IOP‐determined tremor severity in response to essential tremor medications

Reliable evaluation of tremor severity is critical to test therapies in animal models. In essential tremor, primidone, propranolol and ethanol all have reliable therapeutic effects in both patients and tremor mouse models.^31^, ^32^, ^33^ Interestingly, carbamazepine has no clinical benefit to essential tremor patients but has been reported to suppress mouse tremor.^34^ This paradox leads to criticism of tremor animal models and creates substantial obstacles to evaluating new therapies for essential tremor in animal models.

**FIGURE 5:**
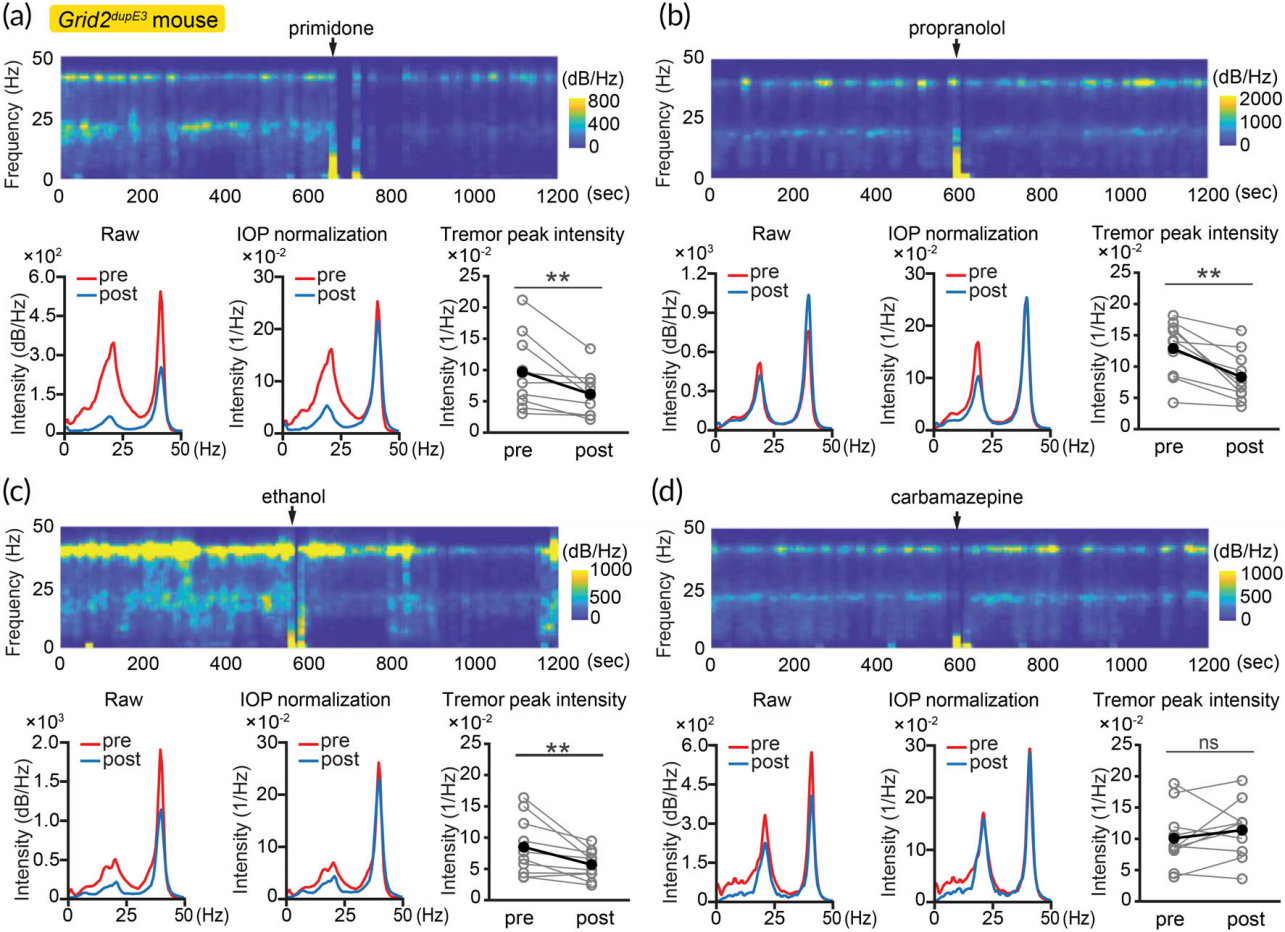
Application of IOP‐based method to test medications for *Grid2* ^*dupE3*^ mouse tremor. (a‐c) Tremor severity reduction after primidone, propranolol, or ethanol administration. (d) Carbamazepine effect. Tremor intensity and locomotor activity reduced proportionally, therefore true tremor severity did not reduce after carbamazepine administration. (*n* = 10 mice) **p < 0.01, Wilcoxon signed‐rank test. Black dots: mean

To validate whether IOP‐determined tremor severity can be used for evaluating therapy for essential tremor, we applied clinically used medications for essential tremor to *Grid2* ^*dupE3*^ mice, including primidone, propranolol and ethanol. We compared IOP‐normalized tremor intensity, which reflects tremor severity, before and after medication administration. We found that medications for essential tremor may reduce overall locomotor activity detected by IOP; yet, the tremor reduction effects still outweighed the locomotor sedative effects after IOP‐normalization (mean(SEM) before medication, mean(SEM) after medication: primidone 0.10 (0.02), 0.06 (0.01); propranolol 0.13 (0.01), 0.08 (0.01); ethanol 0.08 (0.01), 0.06 (0.01); unit: 1/Hz; Figure 5a–c), demonstrating therapeutic effects consistent with clinical observations.

In contrast, the application of carbamazepine, an anticonvulsant that does not have therapeutic benefit to essential tremor patients, showed that tremor reduction was proportional to IOP‐detected locomotor reduction and was purely accountable by locomotor sedative effect (mean(SEM) before medication, mean(SEM) after medication: carbamazepine 0.10 (0.02), 0.11 (0.01); unit: 1/Hz; Figure 5d). Proper normalization with IOP‐based method identified the lack of therapeutic efficacy of carbamazepine, consistent with the clinical observations. Additionally, these results are consistent with the analysis after background subtraction from fitting component (mean(SEM) before medication, mean(SEM) after medication: primidone 0.07 (0.02), 0.04 (0.01); propranolol 0.10 (0.01), 0.06 (0.01); ethanol 0.06 (0.01), 0.04 (0.01); carbamazepine 0.07 (0.01), 0.08 (0.01); unit: 1/Hz; Figure S9). Taken together, force plate‐based IOP method can be applied for better drug screening for the therapeutic effects in mouse tremor.

### 2.6 IOP‐based method detects optogenetically induced rhythmic movement in intact motor circuit

**FIGURE 6:**
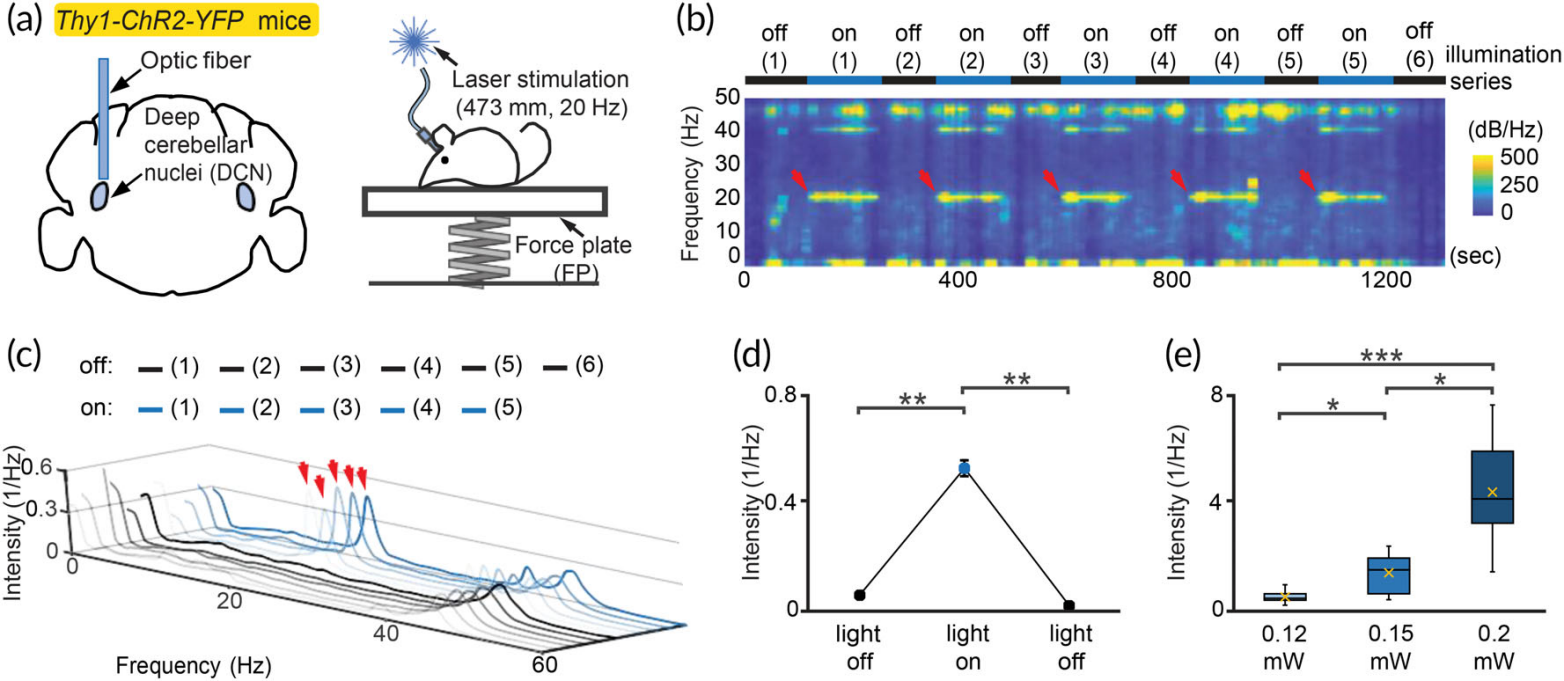
Application of IOP‐based method in the studies of rhythmic motion at 20 Hz. (a) Schemes of optic fiber targeting DCN of non‐tremor *Thy1‐ChR2‐YFP* mouse (left) and optogenetic‐induced rhythmic movement measurement (right). (b, c) The representative analysis of induced 20 Hz rhythmic movements, indicated by red arrows. (d) Significantly increased rhythmic movements during illumination‐on period, compared with adjacent‐off periods. Error bar (SEM) is invisible if smaller than dot. (*n* = 10 tests from a mouse) **p < 0.01, Wilcoxon signed‐rank test. (e) Dependency of induced rhythmic movements on illuminating power. Orange cross: mean. Whiskers: maximum/minimum. (*n* = 10 tests for each power from another mouse) $\chi_{2}^{2}$ = 20.689, *p* < 0.0001, Kruskal–Wallis rank sum test; post hoc: *p < 0.05, ***p < 0.001, Dunn's all‐pairs test with Holm's adjustment

Although IOP‐based method can be reliably applied to measure tremor in *Grid2* ^*dupE3*^ mice with abnormal cerebellar pathology,^24^ it is unclear whether IOP‐based method can also be applied to normal brain circuits for rhythmic or patterned movement. To address this issue, we optogenetically stimulated deep cerebellar nuclei (DCN) in wild‐type mice (Figure 6a).

When applying 20 Hz laser illumination to stimulate DCN, mouse body shaking can be observed during the light‐on period of rhythmic illumination (Movie S3). Spectral analysis also exhibited this 20 Hz rhythmic movement during the light‐on periods, but not in the light‐off periods (mean(SEM): first light‐off 0.06 (0.01), light‐on 0.53 (0.03), second light‐off after 0.05 (0.003); unit: 1/Hz; Figure 6b–d). The harmonic frequency (~40 Hz) was also observed, consistent with previous studies^35^, ^36^, ^37^ and reinforced the benefit of tunable IOP frequency to avoid harmonics. With the fixed power of illumination, the intensity of optogenetic‐induced rhythmic movement showed correlation with the intensity of intrinsic oscillations (Figure S10), indicating that IOP‐based normalization is valid for cerebellar‐dependent rhythmic movement.

**FIGURE 7:**
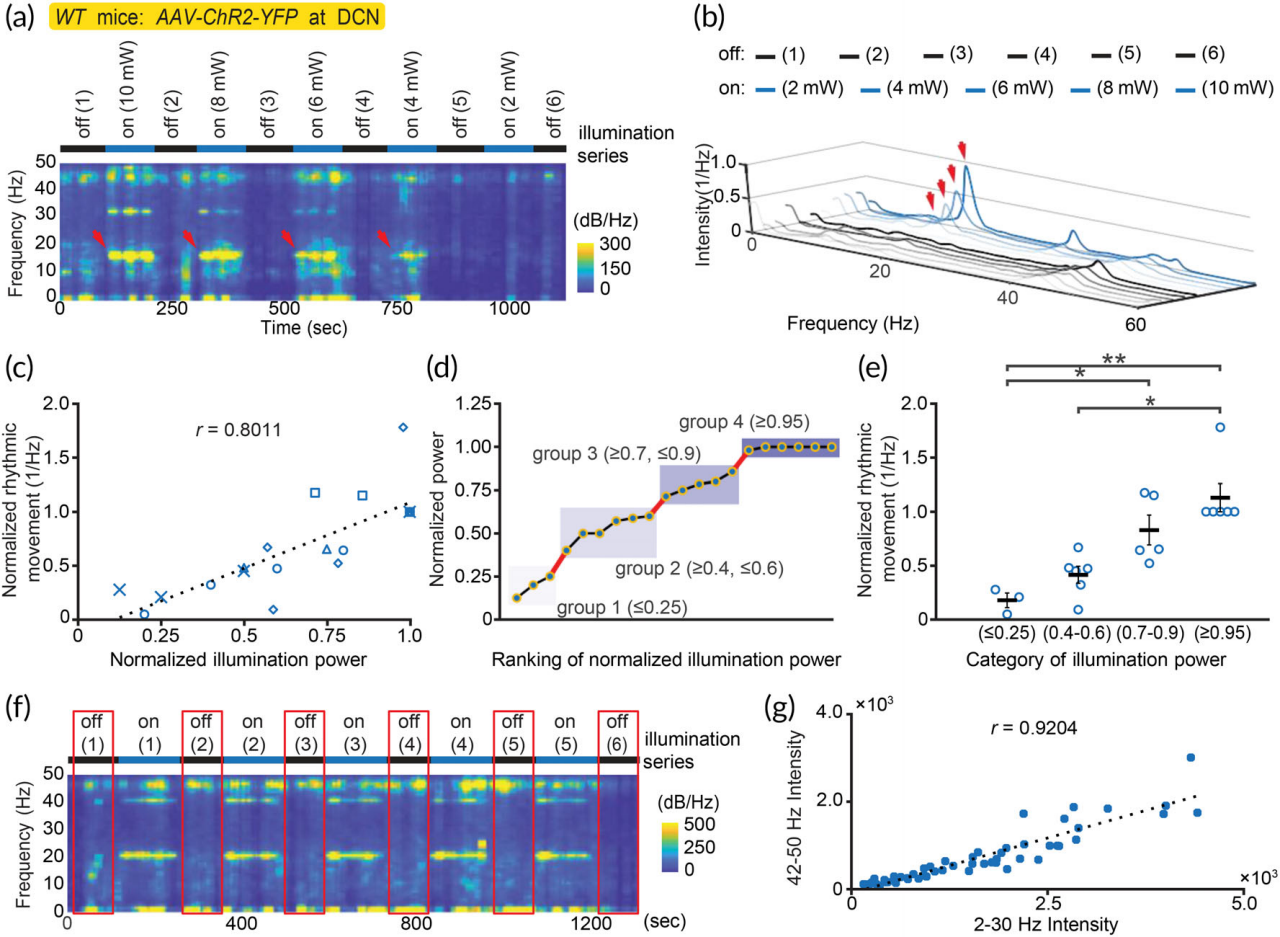
Application of IOP‐based method in the studies of rhythmic motion at 16 Hz and nonrhythmic natural movement. (a, b) The representative analysis of induced 16 Hz rhythmic movement in *WT* mouse with AAV‐mediated channelrhodopsin expression in DCN. Induced rhythmic movements upon different illuminating powers (2–10 mW) are indicated by red arrows. (c) Correlation between normalized rhythmic movement and normalized illuminating power. Illuminating power was normalized to the maximally applied intensity in each recording. Recordings are indicated by different symbols. Data in Figure 7d,e were derived from Figure 7c. (*n* = 20, five recordings from four mice, 3–5 tests per recording.) 95% confidence interval: [0.5555, 0.9181]; *t*_18_ = 5.6785, *p* < 0.0001, *t*‐test. (d) Categorizing the normalized powers based on ranking profile. Each group is distinguished from the adjacent one with the steep gap (indicated red line). (e) Dependency of induced rhythmic movement on illuminating power. Horizontal black bar: mean. Error bar: SEM. $\chi_{3}^{2}$ = 14.068, *p* < 0.01, Kruskal–Wallis rank sum test; post hoc: *p < 0.05, **p < 0.01, Dunn's all‐pairs test with Holm's adjustment. (f) The representative analysis of nonrhythmic natural movements (red rectangles: selected periods). This representative data are identical to Figure 6b. (g) Correlation of intrinsic oscillations and non‐rhythmic natural movement. (*n* = 54; 6 ‐off periods in each of 9 recordings from the mouse shown in Figure 6e) 95% confidence interval: [0.8661, 0.9532]; *t*_52_ = 16.9756, *p* < 0.0001, *t*‐test

Since the strength of illumination can be well defined, we then applied different power of optogenetic stimulation and measured the intensity of induced rhythmic movement at the targeted frequency. It is clear that the intensity of rhythmic movement increased with the illuminating intensity (mean(SEM): 0.12 mW 0.49 (0.07), 0.15 mW 1.38 (0.22), 0.20 mW 4.37 (0.65); unit: 1/Hz; Figure 6e). Consistently, the rhythmic movement shifted to 16 Hz after changing the stimulating frequency to 16 Hz, and the intensity of this rhythmic movement is also modulated by the stimulating strength (mean(SEM): ≤0.25 mW 0.18 (0.07), 0.4–0.6 mW 0.41 (0.08), 0.7–0.9 mW 0.83 (0.14), ≥0.95 mW 1.13 (0.13); unit: 1/Hz; Figure 7a–e). These results suggested that IOP method can be used to study cerebellar‐dependent, optogenetic‐induced rhythmic movement.

### 2.7 IOP in quantifying nonrhythmic natural movement

The next question is whether IOP methods can also be used to study non‐rhythmic movement? We thus examined IOP methods in measuring overall momentum of nonrhythmic, natural movement. We analyzed the light‐off periods in the above‐mentioned optogenetic experiments (Figure 7f). In these periods, mice showed their physiological movement in freely moving state without obvious brisk activity whose motion spectrum may fall into higher frequency ranges. We therefore integrated the 2–30 Hz data to represent the overall momentum. Our data showed a strong correlation between the overall momentum and the intensity of intrinsic oscillations at 42–50 Hz (Figure 7g). The data suggested that IOP can be applied to quantify overall momentum in both rhythmic and nonrhythmic movement. Therefore, kinematics studies targeting specific brisk movement (e.g., reaching for food) may be isolated and separable by overall momentum represented by the IOP‐specific frequencies, which are tunable in the system to fit different experimental scenarios (Figure 1c,d).

## 3 DISCUSSION

In this study, we identified the IOP method, which used intrinsic oscillations of a force plate to detect and quantify the overall momentum due to locomotion. Using tremor as a model, head‐to‐head comparison among IOP‐, video‐ and accelerometer‐based tremor quantification revealed that IOP method can provide more consistent results in terms of tremor severity and frequency stability. IOP method allows quantification of tremor therapeutics with coexisting locomotor suppression and solved the paradoxical results of carbamazepine, suggesting carbamazepine leads to locomotor sedative effect rather than true tremor suppression. IOP method is also applicable to quantify optogenetically induced rhythmic movement. More importantly, it is capable of evaluating overall momentum in nonrhythmic natural movement. In summary (Figure S11), IOP‐based method can detect overall momentum with high temporal resolution, which may facilitate future kinematics studies of a targeted movement that often requires proper adjustment based on overall momentum of the animal.

A few limitations are conceivable in this study. To validate a new method, we focused on rhythmic movement for its clear‐cut readout and known frequency features. While we demonstrated that IOP can reflect momentum in nonrhythmic movement (Figure 7g), further studies are required to optimize IOP usage in other kinematic tasks such as gait or reaching movement. Second, we recorded the video with the settings of 8‐bit color depth and 720p high‐definition (HD) at 60 frame‐per‐second (FPS). While the sampling rate is sufficient to describe 20 Hz tremor, increasing the pixel numbers per frame could improve the resolution of tremor amplitude and potentially improve the tremor measurement. Notably, a 10–15 min not compressed video (required for best tracking and labeling) in our current settings already exceeds 1.0 GB in file size. Further increase of the pixel number or sampling rate will increase the file size and the demand of proper illumination.

Here, we validated IOP as an additional method for kinematics study. Combination of IOP and other methods can be beneficial. Although accelerometer‐based tremor measurement in our setup seems inferior to force plate‐based method, it could be due to a single accelerometer that detects the targeted motion, but not the overall momentum as the force plate measurement does. By combining the two methods, multiple miniature accelerometers attached to the head and limbs could provide detailed kinematics at multiple joints *locally*, and the coexisting IOP method could reflect *global* momentum and frequency integration of coordinated movement. Video is a nonreplaceable method to capture the moving types (e.g., grooming vs. walking). Combination of all methods may provide more comprehensive information for studying kinematics of movement.

In addition to the marker‐less video capturing and accelerometer‐based measurement, locomotion analysis has been performed with the aid of other techniques including skin‐marker (optical marker) tracking system, fluoroscope, and ultrasound‐based motion tracking.^15^, ^16^, ^17^, ^18^, ^19^, ^23^ Skin‐marker tracking systems have been used in human and rodent gait analysis and have an advantage in marking joint position and angles.^15^, ^16^ However, there is also evidence that marker‐based tracking systems can yield large error in estimated kinematics when applied to freely moving rodents.^18^ Besides, marker‐based video capturing may interfere with physiological behavior due to the marker placement and may reduce the versatility of pose estimation and kinematics analysis.^16^ On the other hand, fluoroscope‐based motion tracking can obtain internal bone structure with x‐ray, which is more precise in tracking limbs kinematics compared with traditional video capturing.^18^, ^19^ However, animals have to be relatively fixed or restrained for this tracking technique, which may disturb their natural locomotion and the underlying neuronal activity. In addition to fluoroscopy, ultrasound has also been applied to motion tracking. It is relatively safe in comparison to the possible radiotoxicity that may be caused by fluoroscopy. This new technique has been used in tracking tendons,^23^ and may have the potential of tracking fine movements in rodents. Nevertheless, such technique may also require the animal to be restrained while being scanned, and its anisotropy problem still awaits solutions.^23^ Last but not least, although these techniques perform superiorly in motion tracking of specific limbs or finer structures, they are not designed to track overall motion kinematics, which is the gap that can be filled by our proposed IOP approach.

To solve the difficulty concerning the tracking of global motion kinematics, there have been ample studies on tracking multiple body parts at the same time. These approaches include the application of inertial sensors,^38^, ^39^ marker‐based tracking,^40^ and marker‐less model tracking.^41^ These novel approaches perform well on multiple body parts tracking and thus are good at capturing the whole‐body postures. However, the dispersed nature of multiple body parts tracking still impedes it from integrating the diverse kinematics of each tracked body part into a single index of global motion kinematics. Besides, most of these multiple body parts tracking approaches rely on video capturing,^40^, ^41^ rendering them limited by the temporal and spatial resolutions problems as well as restrain‐related issue confronting video‐based tracking methods. Others rely on innovative inertial sensors tracking, but this approach requires researchers to design and build the sensor system on their own without a well‐established and easy‐to‐use protocol at present.^38^ Hence, optimizations are indispensable for these approaches to be used in global motion kinematics tracking. In comparison, our IOP approach using a force plate is relatively convenient to use, and is able to generate a single index for quantifying global motion and cater to the need of tracking in a timescale of milliseconds.

Additionally, to study the pathophysiology of motion disorders using freely moving animal models, it is imperative to determine whether symptom modulation is resulted from dampening the neuronal mechanisms responsible for the symptom (e.g., tremor generator in the central nervous system) or simply from a reduction of overall activity. Hence, constrained experimental scenarios, such as head‐fixation or physical restraint, may affect action and therefore the expression of motor symptoms. In this context, IOP may provide a more natural way to record and calibrate motor deficits in a freely moving setting and thus yield higher ecological validity for studying neural mechanisms and therapeutics of motion disorders in humans.

Although our study has been performed on animal models, the force plate‐based IOP approach also shows potential in translational human applications. Currently, force plate has been applied to evaluate balancing functions for physical therapy and for identifying individuals at risk of falling.^42^, ^43^ Assessment of neuromusculoskeletal function while standing or walking can also be performed on a force plate for patients with foot and ankle pathology.^44^ With proper tuning of these clinical‐used force plates, our IOP approach can further improve the quantification of these symptoms' severity. Furthermore, it may aid in the evaluation of motion disorders beyond existing applications. For example, IOP calibration of motor symptoms severity can be expanded to assessment of disorders like myoclonus, dystonia, and various types of tremor in the future. Moreover, symptom assessment is not only important for diagnosis of motion disorders but also crucial to evaluation of therapeutics. Hence, in addition to the applications in animal motor phenomena studies, our IOP approach also has the potential of clinical usage.

As demonstrated, IOP can be a useful approach to study tremor pathophysiology. Essential tremor, characterized by action‐dependent tremor, is the most common movement disorder with unsatisfactory pharmacological therapies and poorly understood pathophysiology. As a result, essential tremor has been the model disease used for testing modern therapeutic technology, including deep brain stimulation and magnetic resonance‐guided focus ultrasound.^45^, ^46^, ^47^, ^48^ Therefore, it is pivotal to establish a standard to evaluate the severity of tremor, which should be pathophysiologically related but independent of the highly variable motion states. Current studies have built up the standard protocols for clinical assessments to identify postural and action tremor.^49^, ^50^ However, calibration of tremor intensity in the context of global motor activity modulation requires further effort. Since force plate can faithfully and sensitively detect the force made by local muscular vibration, such as tremor, and IOP can summarize the overall motion of an indicated movement, we suggest that our method can be used to estimate the severity of motion disorder in a specific part of the body by comparing the force/energy imposed by the symptom to the overall force/energy arising from volitional locomotion. Because we normalized the intensity of motion disorder to the overall motion, this method can provide an objective index in the face of the heterogeneity in human patients' symptom expressions.

In summary, the IOP approach is a low‐cost and easy‐to‐use method for evaluation of motor phenomena and motor deficits. Using action tremor as an example disease model, this approach is shown to be capable of capturing the overall motion kinematics that modulate the expressed intensity of motor deficits. By taking advantage of its flexibility to adapt to various recording scenarios, the applications of the IOP method may be expanded to assessments of more motor phenomena and shed new light on human motion disorders in the future.

## 4 CONCLUSIONS

In summary, we simultaneously compared force plate‐based, accelerometer‐based, and video‐based detection of motion in freely moving mice. We demonstrated that the IOP of a force plate provides a more consistent measurement for the overall momentum of locomotion. A reliable method for measuring the overall activity is particularly critical to study the modulators of locomotion. For example, movement changed due to medication effect and neuronal circuitry activation. Combining detections of overall and targeted locomotion will advance the studies in behavior changes of freely moving mice in response to different modulatory elements that instantly affect neuronal circuitry.

## 5 MATERIALS AND METHODS

Details are referred to Supplemental Methods S1.

### 5.1 Animals and surgery

Wild‐type, *Grid2* ^*dupE3*^ strain,^24^ and *Thy1‐ChR2‐YFP* mice were used. All experiments were performed under the protocols approved by the IACUCs at NTU (B202000003) and CUIMC (AAAT7472). The connector for accelerometer was secured on the mouse skull. Optic fiber was implanted to the deep cerebellar nuclei (DCN; AP, −6.24 mm from bregma; ML, +2.1 mm from midline; DV, −1.9 mm from dura). AAV9‐hSyn‐eNPAC 2.0‐WRPE was injected into the DCN.

### 5.2 Force plate assembly

A force plate with IOP (Figure S1) was assembled by three components: a basal plate to support the free‐moving mouse; a loading cell to provide both elasticity for spring effect and linear force‐voltage transformation; and a preamplifier to transmit stable voltage readout (Figure S1b,c). The load cell transfers the weight into voltage at the ratio of 33 mV per gram‐gravity and the gain of the preamplifier is set to 1000×. Thus, the temporal dynamics of the applied force or motion kinematics is transformed into continuous voltage output (Figure S1d and Movie S1). With the existence of IOP, the energy given to the force plate becomes the source of oscillations and is further transformed into the oscillatory power at the intrinsic oscillatory frequency (Figure S1d) by the innate physical property of the force plate. Therefore, the dynamics of the power at this single frequency (IOP) could represent the dynamics of overall motion kinematics, while the spectral distribution of motion can be further deconstructed into different movement types (Figure S1e).

### 5.3 Mechanical tapping

Motion was generated by mechanical tapping on the force plate. Specific frequencies (5 and 7 Hz) were applied with the aid of metronome (Pro Metronome, EUMLab). To demonstrate that the force plate obeys the physical principle of IOP, objects with six different masses (0, 90, 190, 390, 590, and 880 g) were applied to the force plate and random tapping with varying force was applied. The resulting IOP frequency values were used for deriving the reciprocals of square of frequency (1/frequency^2^). The relationship of these values with the corresponding weights was fitted by a linear curve and followed by correlation analysis.

### 5.4 Tremor measurement settings and tremor recordings

While a mouse was freely moving on the force plate, accelerometer signals sampled at 1000 Hz and videos captured at 60 frame‐per‐second were recorded simultaneously. Mouse activity on the force plate was transduced into voltage signal at 1000 Hz. Headstage containing accelerometer was connected to the implant on the mouse's skull. Mouse's body center was determined by using DeepLabCut video processing.^51^

### 5.5 Accelerometer‐based recording

Custom headstage containing an ADXL335 accelerometer (Analog Devices) was connected to the implant adhered on the mouse's skull during the recording. The accelerometer is three‐dimensional and can measure acceleration with a minimum full‐scale range of ±3 g. The sensor is a polysilicon surface‐micromachined structure providing a resistance against acceleration forces. Deflection of the structure is measured using a differential capacitor, resulting in a sensor output, whose amplitude is proportional to acceleration. Phase‐sensitive demodulation techniques are then used to determine the magnitude and direction of the acceleration. Signals were recorded and processed by Cerebus Neural Signal Processor (Blackrock Microsystem) and were digitized at 30 kHz.

### 5.6 Video‐based recording and tracking

Video recording was performed with uEye camera (IDS Image Developing Systems) from an above view. The video was recorded with the resolution of 1280 × 720 pixels, covering the open field of 28 cm × 16 cm, giving the pixel resolution of 0.22 mm in both X and Y direction. The frame rate was 60 frame per second (FPS). The mouse body center was identified by the free algorithm DeepLabCut.^51^ Frame‐by‐frame displacement was used to calculate the mice's moving velocity with the X and Y coordinates of the body center. The velocity data were then transformed into PSD data and subjected to further analyses.

### 5.7 Signal preprocessing for power spectrum analysis and peak determination

Signals from force plate‐based, accelerometer‐based, and video‐based recording were aligned with a manually triggered analogue signal. This signal was sent to the electrophysiological system and lighted up a red LED at the same time. The electrical signal aided in aligning the time frame of force plate‐ and accelerometer‐based recordings. The red LED was set at the margin of the video, aiding in the alignment of video recordings to the electrical signals. The aligned signals were analyzed by custom‐written code in MATLAB.^24^, ^52^, ^53^ Coordinates data from the video‐based tracking, vector magnitudes from the accelerometer recording, and electrical signals from the force plate, were used for power spectrum analysis. Peak value in the power spectrum density (PSD)‐frequency plot was determined by locating the local maximum of concave downward feature.

### 5.8 Signal normalization

In force plate measurement, tremor PSD was normalized to summed PSD amplitude between 40 and 50 Hz (IOP of force plate) in the same period. For video data, tremor PSD data derived from moving velocity was normalized to general activity (overall 2–30 Hz PSD also derived from moving velocity) or moving velocity *per se*. PSD data derived from acceleration was normalized to general activity (overall 2–50 Hz PSD also derived from accelerometer) or moving velocity from video data. To avoid biased value due to small denominator (moving velocity), PSD data of a time point were ruled out if the corresponding moving velocity was not over the threshold of 0.4 mm/s.

### 5.9 Background component subtraction

Two‐term exponential fitting (MATLAB function fit) was applied on the accelerometer spectral data. To avoid the influence of noise and tremor signals on fitting, signals at low‐frequency band (0–5 Hz) and tremor frequency band (13–27 Hz) were excluded. Then corrected spectrum was obtained after subtracting the fitting result from the original spectrum. For background subtraction in force plate data, pchipinterp fitting (MATLAB function fit) on the spectrum excluding tremor frequency band (12–27 Hz) was performed and the result was subtracted from the original spectrum. For video data, one‐term exponential fitting (MATLAB function fit) was applied, with low‐frequency band (0–5 Hz) excluded.

### 5.10 Medication in homozygous *Grid2* ^*dupE3*^ mice

Primidone (1.7 mg/kg), propranolol (10 mg/kg), ethanol (0.49 g/kg), or carbamazepine (30 mg/kg) was applied via intraperitoneal injection.

### 5.11 CV analysis, correlation analysis, and statistics

CV was applied to raw, normalized, and background‐subtracted data. Correlation analysis was applied to PSD in tremor frequency and IOP. For other statistical analyses, Wilcoxon signed‐rank test, Kruskal–Wallis rank sum test, Dunn's all‐pairs test, and Friedman test were performed in RStudio.^54^

### 5.12 Optogenetic stimulation and the analysis of rhythmic movement induction

Optogenetic stimulation was given via a diode laser module with the indicated frequency and illuminating power (see Supplemental Methods S1). The induced motions were recorded on the force plate. PSD data of light‐on or light‐off period were normalized to IOP. The mean PSD of each illuminating strength under light‐on condition was calculated. The correlation of IOP and nonrhythmic natural movement during light‐off period was also analyzed.

## AUTHOR CONTRIBUTIONS

**Chun‐Lun Ni:** Data curation (equal); formal analysis (equal); investigation (equal); methodology (equal); visualization (equal); writing – original draft (equal). **Yi‐Ting Lin:** Data curation (equal); formal analysis (equal); investigation (equal); methodology (equal); software (equal); visualization (equal); writing – original draft (equal). **Liang‐Yin Lu:** Data curation (equal); methodology (equal); software (equal); validation (equal). **Jia‐Huei Wang:** Data curation (equal); methodology (equal); software (equal); validation (equal). **Wen‐Chuan Liu:** Data curation (equal); methodology (equal); software (equal); validation (equal). **Sheng‐Han Kuo:** Funding acquisition (equal); project administration (equal); resources (equal); supervision (equal); writing – review and editing (equal). **Ming‐Kai Pan:** Conceptualization (equal); funding acquisition (equal); investigation (equal); methodology (equal); project administration (equal); resources (equal); software (equal); supervision (equal); writing – review and editing (equal).

## CONFLICT OF INTEREST

The authors have no conflict of interest to declare.

### PEER REVIEW

The peer review history for this article is available at https://publons.com/publon/10.1002/btm2.10432.

## Supporting information


**Appendix S1:** Supporting Information

**Movie S1.** Force plate and its response to external force. A force plate assembled by a support plate, a loading cell and a pre‐amplifier and provides weight‐to‐voltage transformation in real time.

**Movie S2.** Video‐based motion detection of a mouse. Video‐based motion tracking of multiple landmarks of mouse position. The yellow dot (body center) is used for further analysis of mouse movement.

**Movie S3.** Induction of rhythmic movement by cerebellar optogenetic stimulation. The rhythmic blue‐light stimulation at the DCN of a *Thy1‐ChR2‐YFP* mouse induced rhythmic movement when the light turned on. The rhythmic movement ceased when the light turned off. DCN, deep cerebellar nuclei.

## Data Availability

Data available in article supplementary material.
