# Supplementary material for: Tracking motion kinematics and tremor with intrinsic oscillatory property of instrumental mechanics
Source: Bioeng Transl Med. 2022 Oct 22;8(2):e10432. doi: 10.1002/btm2.10432 (PMC10013767; doi:10.1002/btm2.10432)
Supplement: Supplementary file 1 — Appendix S1: Supporting Information [file BTM2-8-e10432-s001.pdf]

Supporting Information for

Tracking motion kinematics and tremor with intrinsic oscillatory property of instrumental mechanics

Chun-Lun Ni, Yi-Ting Lin, Liang-Yin Lu, Jia-Huei Wang, Wen-Chuan Liu, Sheng-Han Kuo, Ming-Kai Pan*

*Corresponding author. Email: emorymkpan@ntu.edu.tw

This PDF file includes:

Supplemental Methods

Supplemental Figure 1 to 11

Legends of Supplemental Movie 1 to 3

Other Supplementary Materials for this manuscript include the following:

Supplemental Movie 1 to 3

Supplemental Methods

Animals

Mice of wild-type (C57BL/6), *Grid2^{dupE3}* (005718, The Jackson Laboratory)[1], and *Thy1-ChR2-YFP* (007612, The Jackson Laboratory) background were used. Nine homozygous *Grid2^{dupE3}* mice (five males and four females) were used in the tremor measurement experiment. Ten homozygous *Grid2^{dupE3}* mice (five males and five females for primidone; six males and four females for others) were used in each of the four medication experiments. Two male *Thy1-ChR2-YFP* mice and four wild-type mice (one male and three females) were used in the optogenetic experiment. All animal experiments were performed under the protocols approved by the Institutional Animal Care and Use Committee at National Taiwan University (approval number: B202000003) and at Columbia University (approval number: AAAT7472).

Surgery

For mice underwent the tremor measurement experiment, custom-made electronic Interconnect boards were adhered to the middle line between bregma and lambda. For *Thy1-ChR2-YFP* mice in the optogenetic experiment, to excite channelrhodopsin-2 (ChR2) expressed in their deep cerebellar nuclei (DCN), unilateral optic fiber (200 μm; Thorlabs) was stereotaxically implanted to the left DCN (AP, -6.24 mm from bregma; ML, +2.1 mm from midline; DV, -1.9 mm from dura). For wild-type mice in the optogenetic experiment, AAV9-hSyn-eNPAC 2.0-WRPE (4.40×10^{11} vg/mL, Optogenetics Resource Center, Stanford University, CA) was injected unilaterally into the DCN coordinates described above. A 33GA Hamilton syringe was used to inject total 2.0 μL virus at the rate of 0.2 μL per min. Syringe was not removed until 10 minutes after completion of injection. The optic fiber was then implanted.

All surgery was done with the aid of stereotaxic frame (David Kopf Instruments). Mice were under isoflurane anesthesia and all implants were secured with dental cement (Sun Medical). Post-surgical recovery was held for at least two weeks before the experiments were performed.

Force plate assembly

A force plate was assembled by 3 parts: a base plate, a load cell and a pre-amplifier. A 28 cm x 16 cm base plate is made by acrylic board. A load cell (Cat No. AT8501, Autoda Inc., China) has a maximal weight load at 300 gm, 1 mV/V voltage transformation index, and 33 μV per gram-gravity weight-voltage transformation. A pre-amplifier assembled with amplifier chip (Cat No. INA122P, Texas Instruments, USA) and a tunable resistance to get a final gain of 1000x. The final output of weight-voltage transformation is 33 mV per gram-gravity.

Tremor measurement settings

Each of the nine mice was recorded for three days, with two periods of 13.5 to 15 minutes in each day. Mice were allowed to move freely on the force plate (customized or Convulsion Meter Convuls 1, Columbus Instruments) during the whole recording periods. Accelerometer signals and videos were recorded at the same time with force plate signals. To synchronize the timing of electrical signals and the videos, a manually triggered analogue signal was sent to the electrophysiological system and lighted up a red LED at the same time. The red LED was set at the margin of the video.

Force plate-based recording

We applied a force plate as the platform to detect weight-based/force-based activity. Imposed force on the platform is transduced linearly into voltage signal (141 mV per 32 g of mass per gravity, or 0.45 V per Newton). Signals were recorded and processed by Cerebus Neural Signal Processor (Blackrock Microsystem) and were digitized at 30 kHz.

Accelerometer-based recording

Custom headstage containing an ADXL335 accelerometer (Analog Devices) was connected to the implant adhered on the mouse’s skull during the recording. The accelerometer is three-dimensional and can measure acceleration with a minimum full-scale range of ±3g. The sensor is a polysilicon surface-micromachined structure built on top of a silicon wafer. Polysilicon

springs suspend the structure over the surface of the wafer and provide a resistance against acceleration forces. Deflection of the structure is measured using a differential capacitor that consists of independent fixed plates and plates attached to the moving mass. The fixed plates are driven by 180° out-of-phase square waves. Acceleration deflects the moving mass and unbalances the differential capacitor resulting in a sensor output whose amplitude is proportional to acceleration. Phase-sensitive demodulation techniques are then used to determine the magnitude and direction of the acceleration. The wire connecting to the headstage was hanging loosely on top of the mouse, allowing the mouse to move freely. Signals were recorded and processed by Cerebus Neural Signal Processor (Blackrock Microsystem) and were digitized at 30 kHz.

Video-based recording and tracking

Video recording was performed with uEye camera (IDS Image Developing Systems) from an above view. Videos were captured at the resolution of 1280×720 pixels and the frame rate of 60 frame per second (FPS).

The displacement of mouse's body center was determined by the free algorithm DeepLabCut.[2] Original videos were being constantly checked while labelling to make the labels as precise as possible. In half of the videos, fifteen frames from each of them were picked by the algorithm to be used for training and testing the machine learning model. The value of cutoff was set at 0.8 as suggested by the developers. Other parameters were set as the default values. The trained model was used to analyze all of the videos. Tracking results were output as x and y coordinates of each tracked point in every frame. This data was used for later analysis in MATLAB.

Signal preprocessing and power spectrum analysis

Signals from the force plate and the accelerometer were down-sampled to 1,000 Hz and filtered with 250 Hz low pass filter. The manually triggered signals were used to align the signals from force plate-based, accelerometer-based, and video-based recording. These processes and later analyses were performed by custom-written code in MATLAB.[1,3,4]

Coordinates data from video-based tracking was preprocessed with MATLAB to derive velocity versus time data of the mouse body center. Velocity data was processed with shifting average of a 20-second window to obtain moving velocity. This shifting-average velocity data was used for power spectrum analysis.

For data from the accelerometer recording, vector magnitudes were calculated from the three-dimensional acceleration data. This data was used for power spectrum analysis. As for the data from the force plate recording, the electrical signals were used for power spectrum analysis.

The power spectrum density (PSD) was calculated by MATLAB function pwelch with 1,000-ms Hanning window and 50% noverlap. This analysis was performed with a moving window of 20 seconds across the time axis. The PSD heatmaps were plotted for all three kinds of recordings. Each recording was cut into halves, making a total of twelve periods for each mouse, and PSD-frequency plot of each period was derived from summed PSD values across the time axis.

Peak determination of rhythmic movement

The peak value in PSD-frequency plot was determined by locating the local maximum of concave downward feature between 15 and 25 Hz. If there were more than one peak feature present, the one with frequency value closest to 20 Hz would be designated as the peak. If two features still remained (e.g. one at 18 Hz and the other at 22 Hz), the one with the same frequency as the peak values in the other period and in the whole recording (if could be identified before this step) would be chosen. If there was no such feature, the one with larger summed difference with its neighboring PSD values would be designated as the peak. On the other hand, if peak feature was absent, the frequency with maximum PSD occurring between 15 and 25 Hz was mandatorily assigned as the peak.

Signal normalization

In force plate measurement, tremor PSD of each frequency in a period was normalized to overall PSD between 40~50 Hz (intrinsic oscillatory property of force plate) in the same period.

For video data, tremor PSD data derived from moving velocity was normalized to general activity (overall 2-30 Hz PSD derived from velocity) or moving velocity. To avoid biased value

due to small denominator (moving velocity), PSD data of a time point was ruled out if the corresponding moving velocity was not over the threshold of 0.4 mm/s.

PSD data derived from acceleration was normalized to general activity (overall 2-50 Hz PSD derived from accelerometer) or moving velocity. Velocity threshold were set up as described above.

Background component subtraction

In order to obtain and subtract the background component of exponential decay in the accelerometer data, two-term exponential fitting (MATLAB function fit) was applied on the spectrum. To avoid the influence of noise and tremor signals on fitting, signals at low frequency band (0-5 Hz) and tremor frequency band (13-27 Hz) were excluded. Then corrected spectrum was obtained after subtracting the fitting result from the original spectrum. For background subtraction in force plate data, pchipinterp fitting (MATLAB function fit) on the spectrum excluding tremor frequency band (12-27 Hz) was performed and the result was subtracted from the original spectrum. For video data, one-term exponential fitting (MATLAB function fit) was applied, with low frequency band (0-5 Hz) excluded.

Medication in homozygous *Grid2^{dupE3}* mice

Primidone (1.7 mg/kg, Tocris 0830), propranolol (10 mg/kg, Tocris 0834), ethanol (0.49 g/kg, Sigma-Aldrich E7023), or carbamazepine (30 mg/kg, Sigma-Aldrich C4024) was prepared in saline and applied via intraperitoneal injection. Mice were freely moving on the force plate for motion detection and the recorded motion was analyzed to study rhythmic activity. Motion was recorded for a total of 20 minutes and medications were administered at 10 min. The interval between medications for each mouse was at least one day. For comparing normalized tremor intensity before and after medication, peak values from the ten mice were compared between the pretest and posttest in each drug administration experiment.

Coefficient of variation analysis

To compare the consistency of tremor intensity and tremor frequency determined by different measurement methods, coefficient of variation (CV) was applied. Raw data, normalized data and background-subtracted data were used for CV analysis.

For analyzing consistency of different tremor measurement methods, peak intensity and peak frequency values from PSD-frequency data were subjected to this analysis. Each CV value was derived from twelve periods (first / second half of six recordings) of a mouse for given method and data type.

Correlation analysis for force plate measurement

To inspect the relationship between PSD in tremor frequency range (15-25 Hz) and intrinsic oscillatory property (IOP) frequency range (40-50 Hz), correlation analysis was applied to data from one homozygous *Grid2*^{dupE3} mouse. The dataset consisted of PSD-frequency data from twenty-two different types of periods, including the twelve periods from splitting recordings into halves, six periods from integrating each recording as a single time window, and four periods from picking a 100-second high-activity and a 100-second low activity time window from two recordings.

In optogenetic experiments, correlation analyses for relationship between PSD in rhythmic movement frequency range (15-25 Hz) and IOP frequency range (42-50 Hz) were also performed on the 20 Hz-illumination dataset. The dataset included fifteen trials of 0.2 mW stimulation from one mouse, as well as twenty-five trials of 0.2 mW stimulation and ten trials of 0.12 mW stimulation from another mouse.

Mechanical tapping

To validate the force plate’s capability to reflect the frequency of motion, motion was generated by mechanical tapping on the force plate. Specific frequencies (5 and 7 Hz) were applied with the aid of metronome (Pro Metronome, EUMLab). Validation of frequency was verified by the raw trace of the recording.

To demonstrate that the force plate obeys the physical principle of IOP, six different weights (0, 90, 190, 390, 590, and 880 g) were applied to the force plate and random tapping was applied. The resulting IOP frequency values were used for deriving the reciprocals of square of frequency ($1/\text{frequency}^2$). The relationship of these values with the corresponding weights were fitted by a linear curve and followed by correlation analysis.

Optogenetic stimulation

Optogenetic stimulation was given via a Cobolt 06-01 diode laser module (Hübner Photonics) connected to a data acquisition board (National Instruments) and controlled with customized LabView (National Instruments) codes. Motions induced by optogenetic stimulation were recorded on the force plate.

Three types of repeated on-off schedules with different time windows of light-on and light-off periods were used (140-second on with 100-second off, 320-second on with 280-second off, and 110-second on with 90-second off). The stimulation frequency was 20 Hz for two of the mice and 16 Hz for the other four. To balance between the time window lengths of light-on and light-off periods in the same session, and to rule out the transition periods in case the analysis would be contaminated, the analyzed periods were truncated to ~82-second, ~264-second, and ~70-second light-on or light-off periods for each type of the schedules, respectively.

Analysis: consistency of rhythmic movement induction

Data from one mouse being given two sessions of 20 Hz, 0.2 mW stimulation were analyzed. The sessions were on the 140-100 schedule (see **Optogenetic stimulation** section) and were recorded with five light-on periods and six light-off periods. PSD data of light-on or light-off period was normalized by the area-under-curve of the IOP frequency range. Notice that the IOP range was set as 42-50 Hz to exclude intensity of harmonics around 40 Hz. The mean PSD values in light-on and light-off periods were calculated for each session.

Analysis: dependency of rhythmic movement on stimulation intensity (20 Hz)

One mouse was treated with 0.12, 0.15, and 0.2 mW stimulation at 20 Hz, with two sessions for each strength. Two of six sessions were on the 140-100 schedule and the other four were on the 320-280 schedule (see **Optogenetic stimulation** section). Each session was recorded with five light-on periods and six light-off periods. PSD data was normalized by the area-under-curve of the IOP frequency range. Notice that the IOP range was set as 42-50 Hz to exclude intensity of harmonics around 40 Hz. The mean PSD of each illuminating strength under light-on condition was calculated.

Analysis: dependency of rhythmic movement on stimulation intensity (16 Hz)

To further explore dependency of rhythmic movement on stimulation strength, a panel of stimulation strengths (0.12 mW to 51 mW, for suiting adequate intensity level for different mice) were applied. A 16 Hz frequency that is different from Grid2 tremor at 20 Hz was applied to confirm the specificity of stimulation. Total five sessions with three to five illumination trials per session were recorded. All sessions were on the 110-90 schedule (see **Optogenetic stimulation** section).

Due to the variation of adequate stimulation strengths among mice, the illuminating power was normalized by transforming it to percentage values with the largest one in the given session as 100%. The intensity of rhythmic movement (as normalized PSD values) was also calibrated, with the intensity of movement corresponding to the strongest stimulation as 100% in the given session.

Correlation was analyzed between percentage values of stimulation intensity and percentage values of tremor intensity. To further analyze the dependency of rhythmic movement on illuminating strength, different illuminating strengths were categorized into four groups (≤ 0.25, 0.4-0.6, 0.7-0.9, and ≥ 0.95), based on the steep gaps shown on the ranking plot of illuminating strength. The PSD data from these groups were analyzed statistically.

Analysis: representativeness of intrinsic oscillations for non-rhythmic movement

To investigate whether the intrinsic oscillations of the force plate can reflect non-rhythmic natural movement of a mouse, light-off data (fifty-four periods) from one mouse of *Thy1-ChR2-YFP* background was analyzed. Since freely-moving mice under light-off conditions tend to show their physiological movement without brisk activity, PSD of the frequency range 2-30 Hz were integrated for representing the overall non-rhythmic momentum. Correlation analysis was then performed between the integrated PSD of the IOP frequency range 42-50 Hz and the integrated PSD of the overall non-rhythmic movement frequency range 2-30 Hz.

Statistics

Analyses of tremor measurement data and medication effects from homozygous *Grid2*^{dupE3} mice were performed with nonparametric Wilcoxon signed-rank test. Analyses of background-subtracted data and the comparisons between original data and background-subtracted data were also performed with Wilcoxon signed-rank test. For analyzing rhythmic movement induced by optogenetic stimulation, Wilcoxon signed-rank test was used in the first analysis (20 Hz, fixed illuminating power) to compare the tremor intensity during light-on periods and light-off periods. Kruskal-Wallis rank sum test was used in the other two analyses (different powers at 20 and 16 Hz) to compare among different strengths of stimulation. Dunn's all-pairs test with Holm’s adjustment was used for post-hoc analyses. For comparing multiple methods of accelerometer-based and video-based measurement, Friedman test followed by post-hoc Siegel-Castellan all-pairs test with Holm’s adjustment was applied. For all tests, two-tailed was applied and the significant level was set at $p < 0.05$. Wilcoxon signed rank test, Kruskal-Wallis rank sum test, Dunn's all-pairs test, Friedman test, and Siegel-Castellan all-pairs test were performed with R, operating in RStudio.[5] For all correlation analyses, two-tailed t-tests were applied and the significant level was set at $p < 0.05$. Correlation analyses were performed in GraphPad Prism 8 (GraphPad Software). Corresponding t-tests and calculation of 95% confidence intervals were performed in Microsoft Excel (Microsoft).

Supplemental Figures

Supplemental Figure 1

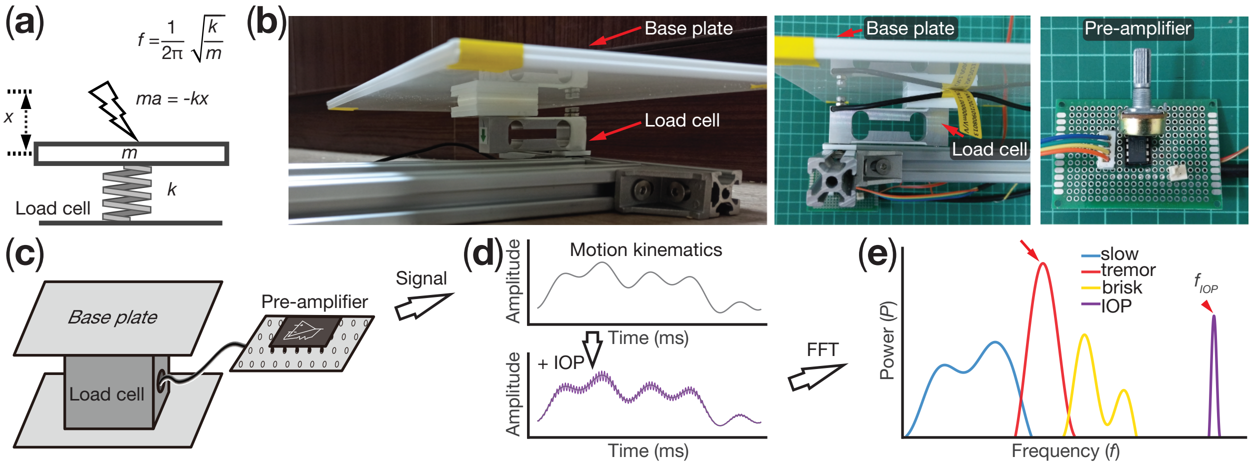

Figure S1. Force plate assembly and its properties to transform motion kinematics into intrinsic oscillations. (**a**) Scheme of force plate-based motion detection with intrinsic oscillatory properties. The oscillatory frequency (*f*) of a force plate depends on system stiffness (*k*) and mass (*m*), but not displacement (*x*) or acceleration (*a*). (**b** and **c**) Force plate assembly and its key components. (**d** and **e**) Processing of data and target feature extraction in the frequency domain. The amplitude and frequency stabilities of tremor peak (e, red arrow) and its relation to overall motion kinematics represented by IOP peak (e, red arrow head) are the key targets evaluated in the later experiments.

Supplemental Figure 2

ACC vs Video: peak intensity

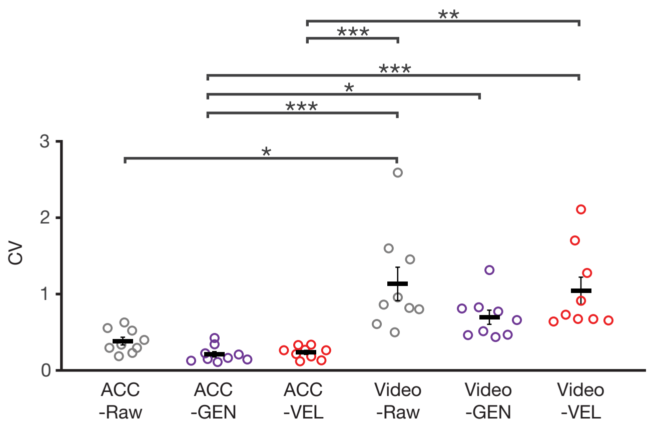

Figure S2. Comparison of tremor peak intensity determined by accelerometer- and video-based methods. Each CV value was derived from the first and second half periods of a recording. Note that all tremor-measuring methods are simultaneously recorded and head-to-head compared. Therefore, **Figure S2** covers the data source of **Figure 2** and **Figure 3** (n = 9 mice). Mean(SEM): ACC-Raw 0.38 (0.05), ACC-GEN 0.21 (0.04), ACC-VEL 0.24 (0.03), Video-Raw 1.13 (0.22), Video-GEN 0.69 (0.09), Video-VEL 1.04 (0.18). $\chi^2_5 = 37.571$, $p < 0.0001$, Friedman test; post-hoc: *$p < 0.05$, **$p < 0.01$, ***$p < 0.001$, Siegel-Castellan all-pairs test with Holm's adjustment. Black horizontal bar: mean. Error bar: SEM. ACC: accelerometer; CV: coefficient of variation; GEN: general activity; VEL: velocity.

Supplemental Figure 3

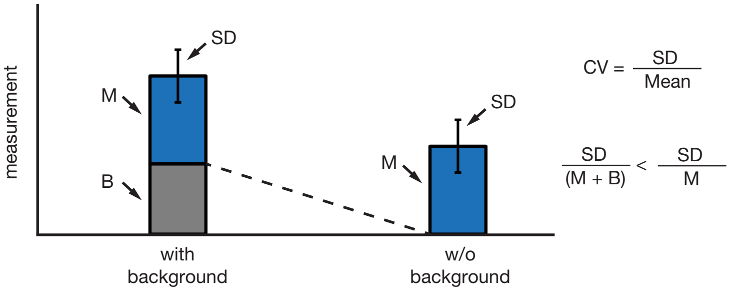

Figure S3. Impacts of background component on CV value. Before removing background component from the measured signal, the overestimated mean value reduces CV value. B: background; CV: coefficient of variation; M: mean; SD: standard deviation.

Supplemental Figure 4

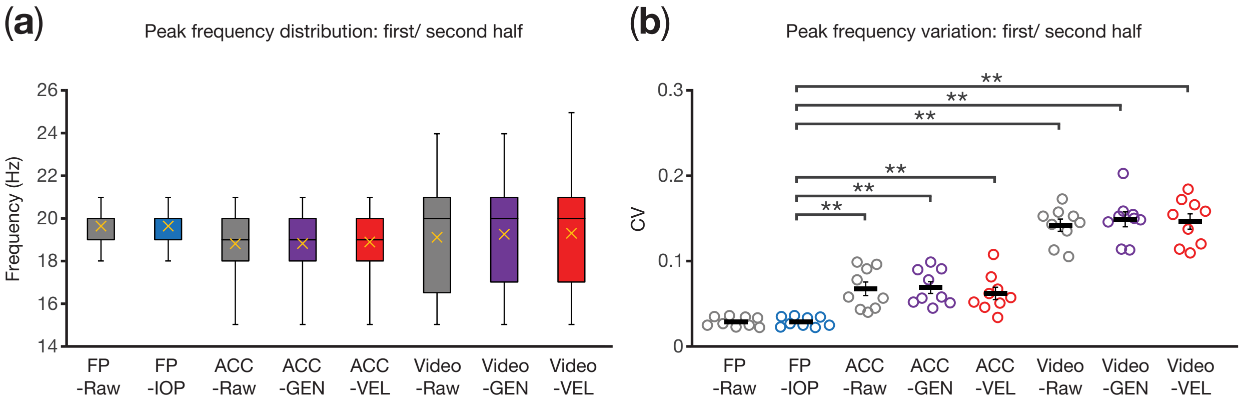

Figure S4. Tremor peak frequency determination by force plate-, accelerometer-, and video-based methods. (**a**) Distribution of peak frequency determined by force plate-based, accelerometer-based, and video-based methods (n = 9 recordings from nine mice). Orange cross: mean. Whiskers: maximum and minimum. (**b**) Comparison of CV of tremor peak frequency determined by force plate-based, accelerometer-based, and video-based methods. Each CV was derived from the first and second half periods of a recording. Mean(SEM): FP-Raw 0.03 (0.002), FP-IOP 0.03 (0.002), ACC-Raw 0.07 (0.01), ACC-GEN 0.07 (0.01), ACC-VEL 0.06 (0.01), Video-Raw 0.14 (0.01), Video-GEN 0.15 (0.01), Video-VEL 0.15 (0.01). Horizontal black bar: mean. Error bar: SEM. (n = 9 mice) $^{**}p < 0.01$, Wilcoxon signed-rank test. ACC: accelerometer; CV: coefficient of variation; FP: force plate; GEN: general activity; IOP: intrinsic oscillatory property; VEL: velocity.

Supplemental Figure 5

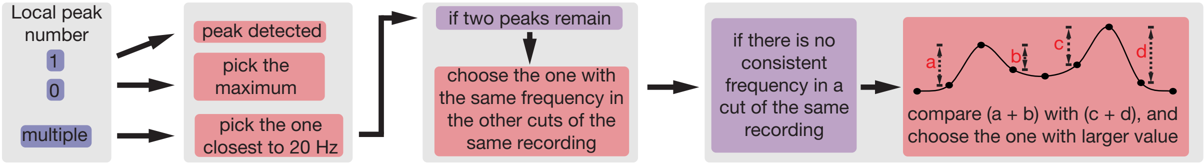

Figure S5. Flowchart of *Grid2^{dupE3}* mouse tremor peak determination with local maximum criteria. A true peak of tremor is assigned if a single concave downward (peak feature) is identified between 15 and 25 Hz. If there is no concave downward identified between 15 and 25 Hz, peak is mandatorily assigned to the frequency with the intensity of local maximum. The algorithm is applied to determine the representative peak if multiple peaks are found in this range.

Supplemental Figure 6

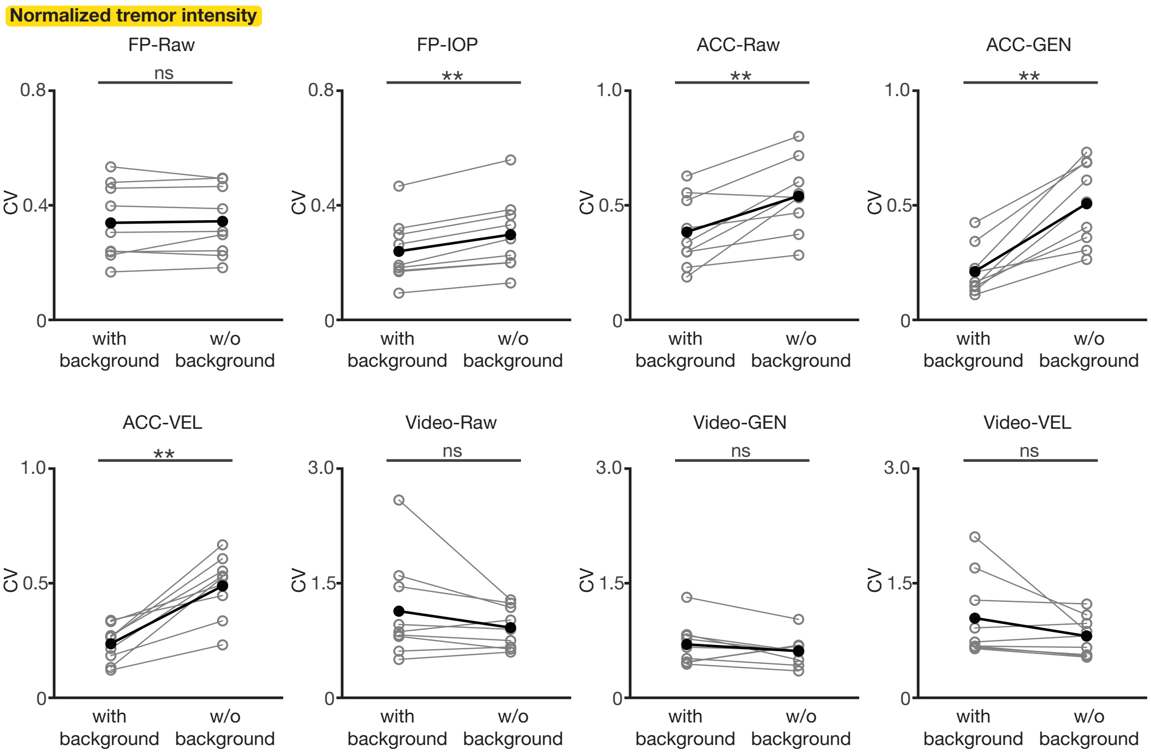

Figure S6. Elevated CV of tremor severity determination after subtracting background components in all accelerometer-based methods. Each CV was derived from the first and second half periods of a recording. Mean(SEM) with background, mean(SEM) without background: FP-Raw 0.34 (0.04), 0.34 (0.04); FP-IOP 0.24 (0.04), 0.30 (0.04); ACC-Raw 0.38 (0.05), 0.54 (0.05); ACC-GEN 0.21 (0.04), 0.51 (0.06); ACC-VEL 0.24 (0.03), 0.49 (0.04); Video-Raw 1.13 (0.22), 0.91 (0.09); Video-GEN 0.69 (0.09), 0.61 (0.06); Video-VEL 1.04 (0.18), 0.80 (0.08). Black dot: mean. (n = 9 mice) $^{**}p < 0.01$, Wilcoxon signed-rank test. ACC: accelerometer; CV: coefficient of variation; FP: force plate; GEN: general activity; IOP: intrinsic oscillatory property; VEL: velocity.

Supplemental Figure 7

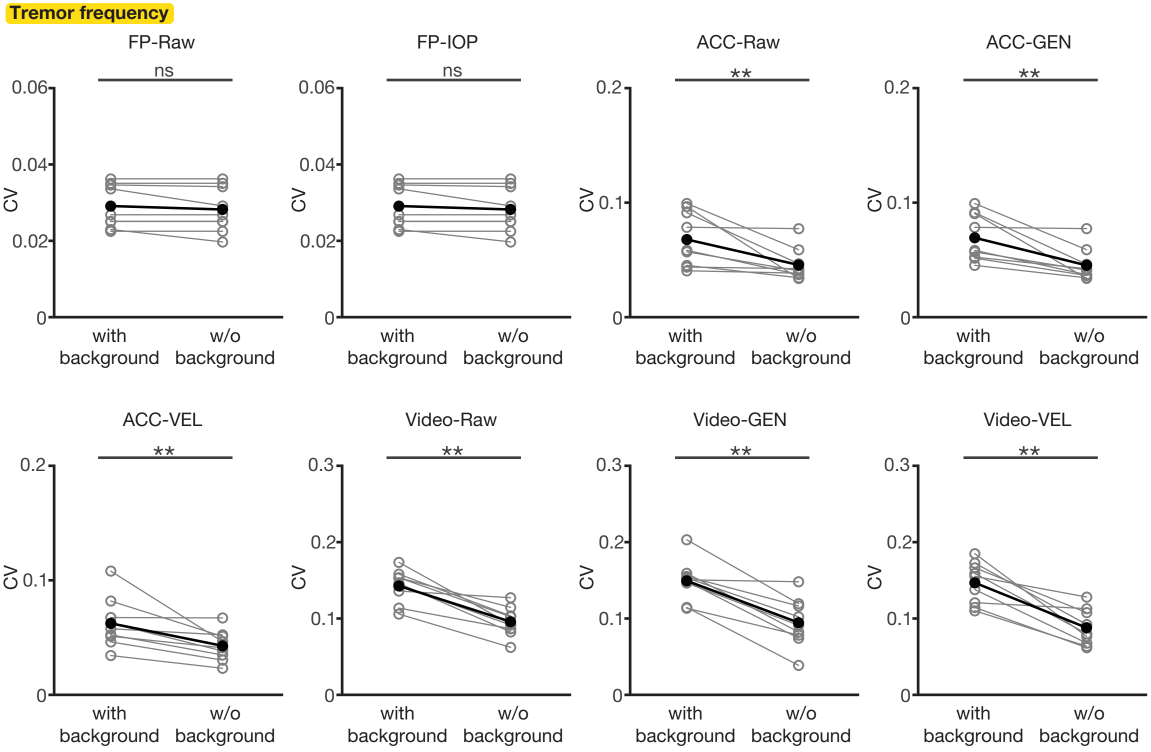

Figure S7. Reduced CV of tremor frequency determination after subtracting background component in all methods. Each CV was derived from the first and second half periods of a recording. Mean(SEM) with background, mean(SEM) without background: FP-Raw 0.03 (0.002), 0.03 (0.002); FP-IOP 0.03 (0.002), 0.03 (0.002); ACC-Raw 0.07 (0.01), 0.05 (0.005); ACC-GEN 0.07 (0.01), 0.05 (0.005); ACC-VEL 0.06 (0.01), 0.04 (0.004); Video-Raw 0.14 (0.01), 0.10 (0.01); Video-GEN 0.15 (0.01), 0.09 (0.01); Video-VEL 0.15 (0.01), 0.09 (0.01). Black dot: mean. (n = 9 mice) *$p < 0.05$, **$p < 0.01$, Wilcoxon signed-rank test. ACC: accelerometer; CV: coefficient of variation; FP: force plate; GEN: general activity; IOP: intrinsic oscillatory property; VEL: velocity.

Supplemental Figure 8

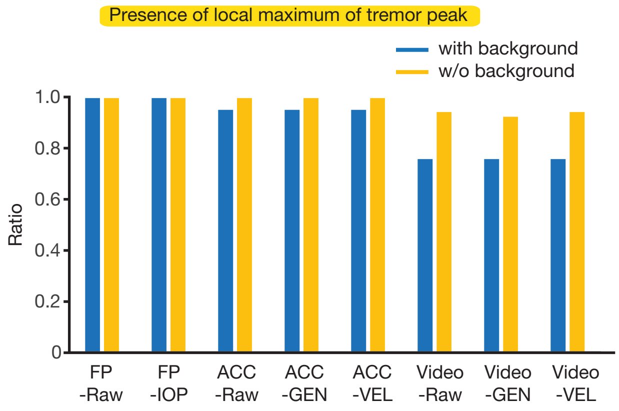

Figure S8. Peak identification ratio in all methods. Ratio of single peak identification. Peak identification process is described in **Figure S5**. Likelihood with background, likelihood without background: FP-Raw 1, 1; FP-IOP 1, 1; ACC-Raw 0.95, 1; ACC-GEN 0.95, 1; ACC-VEL 0.95, 1; Video-Raw 0.76, 0.94; Video-GEN 0.76, 0.93; Video-VEL 0.76, 0.94. FP: force plate. IOP: normalization to intrinsic oscillatory property. ACC: accelerometer; FP: force plate; GEN: general activity; IOP: intrinsic oscillatory property; VEL: velocity.

Supplemental Figure 9

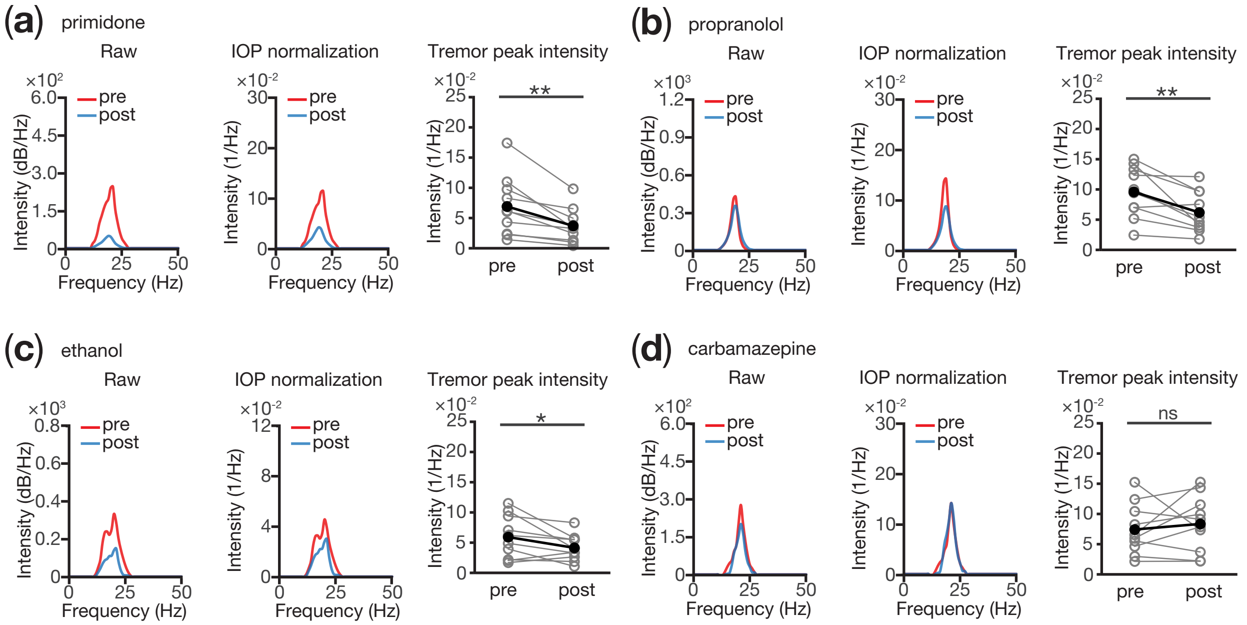

Figure S9. Medication effects on homozygous *Grid2^dupE3^* mouse tremor. Same data source of Figure 5a-d with additional processing of background component subtraction. (**a**-**c**) Tremor severity reduction after administration of primidone (1.7 mg/kg), propranolol (10 mg/kg), or ethanol (0.49 g/kg). (**d**) Proportionally reduced locomotor activity and tremor intensity but not tremor severity after administration of carbamazepine (30 mg/kg). Mouse locomotion was measured 10 minutes before administration via intraperitoneal injection. Mean(SEM) before medication, mean(SEM) after medication: primidone 0.07 (0.02), 0.04 (0.01); propranolol 0.10 (0.01), 0.06 (0.01); ethanol 0.06 (0.01), 0.04 (0.01); carbamazepine 0.07 (0.01), 0.08 (0.01); unit: 1/Hz. Black dot: mean. (n =10 mice) *p < 0.05, **p < 0.01, Wilcoxon signed-rank test. IOP: intrinsic oscillatory property.

Supplemental Figure 10

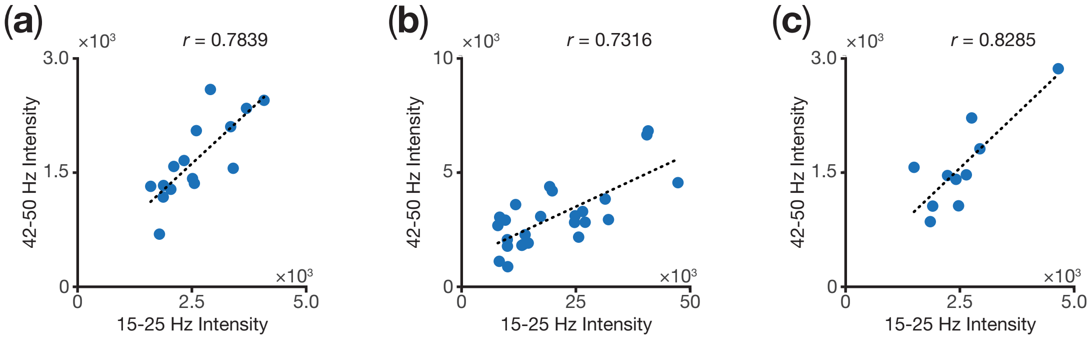

Figure S10. Correlation between intrinsic oscillations and optogenetically induced rhythmic movement. Overall intensity (42 to 50 Hz) regarded as intrinsic oscillations shows correlation with overall intensity (15 to 25 Hz) induced by optogenetic stimulation at 20 Hz. (**a**) Results of 0.2 mW stimulation from a mouse. (n = 15, three recordings, five tests per recording) 95% confidence interval: [0.4539, 0.9248]; $t_{13} = 4.5522$, $p < 0.001$, t-test. (**b**) Results of 0.2 mW stimulation from another mouse. (n = 25, five recordings, five tests per recording) 95% confidence interval: [0.4733, 0.8741]; $t_{23} = 5.1466$, $p < 0.0001$, t-test. (**c**) Results of 0.12 mW stimulation from the same mouse described in B. (n = 10, two recordings, five tests per recording) 95% confidence interval: [0.4157, 0.9583]; $t_8 = 4.1846$, $p < 0.01$, t-test.

Supplemental Figure 11

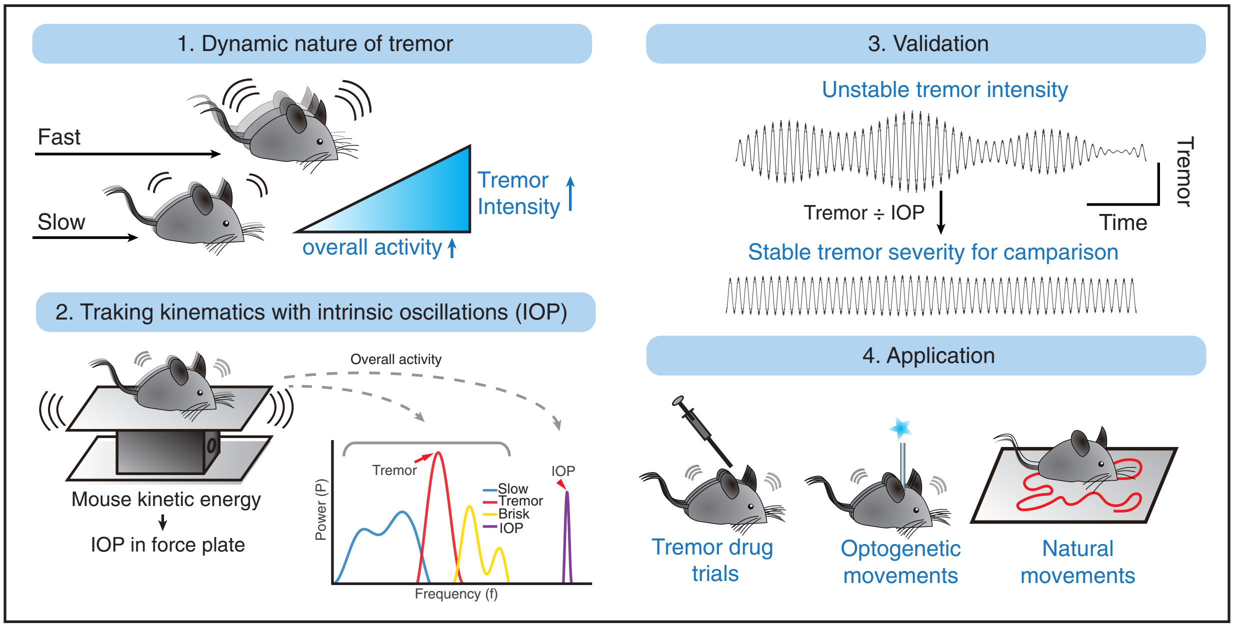

Figure S11. Properties, validation and application of force plate with intrinsic oscillatory property. Tremor intensity varies greatly with overall activity. Using a force plate with intrinsic oscillatory property (IOP), we can transform the kinetic energy into the power of intrinsic oscillations to represent overall activity. Therefore, tremor intensity normalized with IOP can generate a more stable tremor severity, which allows comparison within the same animal with/without interventions or comparison between animals. The IOP method are applied and validated in tremor drug trials, optogenetic movements and natural movements. IOP: intrinsic oscillatory property.

Legends of Supplemental Movie

Movie S1. Force plate and its response to external force.

A force plate assembled by a support plate, a loading cell and a pre-amplifier and provides weight-to-voltage transformation in real time.

Movie S2. Video-based motion detection of a mouse.

Video-based motion tracking of multiple landmarks of mouse position. The yellow dot (body center) is used for further analysis of mouse movement.

Movie S3. Induction of rhythmic movement by cerebellar optogenetic stimulation.

The rhythmic blue-light stimulation at the DCN of a *Thy1-ChR2-YFP* mouse induced rhythmic movement when the light turned on. The rhythmic movement ceased when the light turned off. DCN: deep cerebellar nuclei.

References quoted in this supplemental file

1. Pan MK, Li YS, Wong SB, et al. Cerebellar oscillations driven by synaptic pruning deficits of cerebellar climbing fibers contribute to tremor pathophysiology. *Sci Transl Med*. Jan 15 2020;12(526)doi:10.1126/scitranslmed.aay1769
2. Mathis A, Mamidanna P, Cury KM, et al. DeepLabCut: markerless pose estimation of user-defined body parts with deep learning. *Nat Neurosci*. Sep 2018;21(9):1281-1289. doi:10.1038/s41593-018-0209-y
3. Pan MK, Tai CH, Liu WC, Pei JC, Lai WS, Kuo CC. Deranged NMDAergic cortico-subthalamic transmission underlies parkinsonian motor deficits. *J Clin Invest*. Oct 2014;124(10):4629-41. doi:10.1172/JCI75587
4. Pan MK, Kuo SH, Tai CH, et al. Neuronal firing patterns outweigh circuitry oscillations in parkinsonian motor control. *J Clin Invest*. Dec 1 2016;126(12):4516-4526. doi:10.1172/JCI88170
5. R Core Team. R: A language and environment for statistical computing. *R Foundation for Statistical Computing*. 2018:Vienna, Austria.
